# Supplementary material for: Global burden and trends of ectopic pregnancy: An observational trend study from 1990 to 2019
Source: PLoS One. 2023 Oct 26;18(10):e0291316. doi: 10.1371/journal.pone.0291316 (PMC10602312; doi:10.1371/journal.pone.0291316)
Supplement: S1 Table — (DOCX) [file pone.0291316.s001.docx]

S1 Table. Incidence of ectopic pregnancy in 1990 and 2019 for all locations, with EAPC from 1990 and 2019.

| location | Num_1990 | ASR_1990 | Num_2019 | ASR_2019 | Num_change | EAPC_CI |
| --- | --- | --- | --- | --- | --- | --- |
| Afghanistan | 16.9 (12.7 to 22.5) | 370.38 (277.07 to 500.02) | 45.3 (34 to 60.3) | 269.84 (202.53 to 362.5) | 1.69% (1.4 to 2.01) | -1.15% (-1.28 to -1.02) |
| Albania | 5 (3.6 to 6.9) | 272.96 (197.06 to 368.57) | 2.1 (1.5 to 2.8) | 166.47 (121.31 to 223.15) | -0.58% (-0.63 to -0.52) | -1.78% (-2.1 to -1.45) |
| Algeria | 26.8 (19.5 to 35.8) | 251.83 (183.73 to 339.96) | 41.2 (29.5 to 56.8) | 169.38 (121.62 to 233.55) | 0.54% (0.36 to 0.76) | -0.64% (-1.21 to -0.07) |
| American Samoa | 0.1 (0.1 to 0.1) | 384.22 (284.36 to 518.72) | 0.1 (0 to 0.1) | 234.74 (171.53 to 315.75) | -0.38% (-0.47 to -0.3) | -1.69% (-1.73 to -1.64) |
| Andorra | 0 (0 to 0.1) | 147.44 (107.55 to 204.61) | 0.1 (0 to 0.1) | 144.57 (103.13 to 195.81) | 0.17% (-0.04 to 0.45) | -0.01% (-0.05 to 0.03) |
| Angola | 20.2 (15.2 to 26.9) | 436.7 (331.33 to 585.1) | 43 (32.4 to 58.5) | 300.83 (227.28 to 406.18) | 1.13% (0.86 to 1.37) | -1.22% (-1.36 to -1.08) |
| Antigua and Barbuda | 0 (0 to 0.1) | 125.18 (92.74 to 169.55) | 0 (0 to 0.1) | 87.37 (63.88 to 118.37) | -0.05% (-0.17 to 0.08) | -1.44% (-1.53 to -1.36) |
| Argentina | 74.6 (55.8 to 98.9) | 465.49 (347.47 to 618.37) | 83.6 (61.6 to 113.3) | 352.47 (259.65 to 474.04) | 0.12% (-0.02 to 0.33) | -0.78% (-0.87 to -0.7) |
| Armenia | 4.4 (3.2 to 6) | 232.03 (169.37 to 309.11) | 2.4 (1.7 to 3.2) | 155.76 (114.72 to 207.34) | -0.47% (-0.55 to -0.36) | -0.86% (-1.37 to -0.36) |
| Australia | 4.7 (3.3 to 6.6) | 51.09 (36.71 to 72.28) | 3.6 (2.8 to 4.7) | 30.1 (23.56 to 38.99) | -0.22% (-0.35 to -0.04) | -0.58% (-1.22 to 0.05) |
| Austria | 15.4 (11.8 to 19.9) | 375.02 (289.36 to 478.99) | 15.9 (11.9 to 21.2) | 393.08 (295.25 to 522.52) | 0.03% (-0.15 to 0.26) | -0.02% (-0.25 to 0.2) |
| Azerbaijan | 11.5 (8.4 to 15.6) | 260.8 (192.69 to 350.81) | 8.2 (6 to 11.1) | 149.56 (110.14 to 202.48) | -0.28% (-0.42 to -0.18) | -1.32% (-1.59 to -1.05) |
| Bahamas | 0.2 (0.2 to 0.3) | 132.67 (98.44 to 178.51) | 0.2 (0.1 to 0.2) | 86.53 (63.57 to 115.33) | -0.13% (-0.26 to 0.02) | -1.79% (-1.95 to -1.64) |
| Bahrain | 0.4 (0.3 to 0.6) | 170.11 (125.64 to 232.69) | 0.6 (0.4 to 0.8) | 81.85 (59.88 to 112.8) | 0.33% (0.17 to 0.51) | -2.62% (-2.84 to -2.4) |
| Bangladesh | 149.7 (111 to 202.9) | 275.36 (205.8 to 368.18) | 105.5 (77.6 to 142.9) | 112.19 (83.12 to 151.81) | -0.3% (-0.39 to -0.2) | -2.94% (-3.03 to -2.86) |
| Barbados | 0.2 (0.1 to 0.2) | 109.5 (81.91 to 147.09) | 0.1 (0.1 to 0.2) | 86.5 (63.97 to 116.76) | -0.26% (-0.35 to -0.15) | -0.72% (-0.93 to -0.5) |
| Belarus | 15.5 (11.1 to 20.8) | 299.78 (217.43 to 402.25) | 14.3 (10.1 to 19.6) | 323.57 (229.76 to 442.83) | -0.08% (-0.28 to 0.16) | 1.27% (0.75 to 1.79) |
| Belgium | 11.6 (8.2 to 15.8) | 228.66 (163.06 to 310.58) | 12.6 (9.1 to 17) | 251.62 (180.92 to 337.09) | 0.09% (-0.1 to 0.33) | 0.41% (0.32 to 0.51) |
| Belize | 0.2 (0.2 to 0.3) | 272.3 (205.4 to 358.21) | 0.3 (0.2 to 0.4) | 125.49 (92.96 to 169.92) | 0.24% (0.11 to 0.39) | -2.51% (-2.61 to -2.41) |
| Benin | 9.3 (7.1 to 12.5) | 416.8 (319.53 to 565.05) | 19 (14.2 to 25.7) | 311.07 (233.39 to 415.21) | 1.05% (0.79 to 1.26) | -0.87% (-0.92 to -0.83) |
| Bermuda | 0 (0 to 0) | 94.76 (69.3 to 131.9) | 0 (0 to 0) | 89.7 (64 to 123.95) | -0.25% (-0.39 to -0.09) | 0.01% (-0.27 to 0.29) |
| Bhutan | 0.9 (0.7 to 1.2) | 314.95 (235.89 to 421.02) | 0.6 (0.4 to 0.8) | 127.7 (93.14 to 174.86) | -0.4% (-0.47 to -0.31) | -3.26% (-3.33 to -3.2) |
| Bolivia (Plurinational State of) | 16.1 (12.1 to 21.4) | 537.91 (404.37 to 714.52) | 21.8 (16.2 to 29.2) | 351.68 (261.72 to 471.56) | 0.35% (0.18 to 0.52) | -1.35% (-1.53 to -1.17) |
| Bosnia and Herzegovina | 3.7 (2.7 to 5.1) | 151.7 (109.87 to 208.43) | 1.6 (1.1 to 2.2) | 112.43 (79.32 to 151.82) | -0.57% (-0.65 to -0.47) | -1.15% (-1.58 to -0.72) |
| Botswana | 3.4 (2.6 to 4.4) | 524.44 (395.53 to 672.49) | 4.1 (3.1 to 5.3) | 297.37 (224.68 to 385.61) | 0.22% (0.01 to 0.53) | -1.81% (-1.87 to -1.74) |
| Brazil | 96.1 (73.9 to 125.9) | 117.89 (91.11 to 154.41) | 100.4 (75.3 to 136.5) | 85.44 (64.41 to 115.48) | 0.05% (-0.04 to 0.13) | -0.93% (-1.04 to -0.82) |
| Brunei Darussalam | 0.2 (0.2 to 0.3) | 153.45 (114.2 to 205.16) | 0.2 (0.2 to 0.3) | 83.5 (61.32 to 114.99) | -0.06% (-0.18 to 0.1) | -2.21% (-2.34 to -2.08) |
| Bulgaria | 5.2 (3.7 to 7) | 139.97 (99.56 to 189.07) | 3.6 (2.7 to 4.8) | 135.63 (99.39 to 183.58) | -0.31% (-0.46 to -0.11) | 0.8% (0.45 to 1.15) |
| Burkina Faso | 18.3 (13.9 to 24.6) | 438.13 (334.88 to 587.28) | 35.7 (26.7 to 48.6) | 330.32 (250.08 to 446.31) | 0.95% (0.68 to 1.12) | -1.07% (-1.11 to -1.03) |
| Burundi | 10.6 (7.9 to 14.5) | 432.43 (319.94 to 593.54) | 18.7 (13.9 to 25) | 355.59 (262.37 to 471.35) | 0.77% (0.52 to 1.06) | -0.62% (-0.69 to -0.54) |
| Cabo Verde | 0.4 (0.3 to 0.6) | 268.02 (202.13 to 359.11) | 0.4 (0.3 to 0.5) | 121.89 (91.33 to 165.34) | -0.12% (-0.22 to 0.02) | -2.83% (-2.97 to -2.68) |
| Cambodia | 15 (11.3 to 20) | 302.91 (226.39 to 403.36) | 11.4 (8.3 to 15.7) | 122.51 (89.98 to 168.08) | -0.24% (-0.34 to -0.13) | -3.32% (-3.52 to -3.12) |
| Cameroon | 17.2 (13 to 23.6) | 357.02 (271.86 to 481.43) | 32.9 (24.5 to 45.5) | 218.18 (163.94 to 297.86) | 0.91% (0.72 to 1.12) | -1.41% (-1.66 to -1.16) |
| Canada | 17.3 (12.4 to 23.8) | 110.62 (79.5 to 151.78) | 18.5 (13.2 to 26.4) | 111.18 (80.2 to 158.5) | 0.07% (-0.08 to 0.25) | 0.32% (0.17 to 0.47) |
| Central African Republic | 4.8 (3.7 to 6.6) | 371.4 (280.16 to 499.91) | 7.4 (5.6 to 10) | 275.37 (206.48 to 368.62) | 0.53% (0.32 to 0.75) | -0.92% (-1.01 to -0.82) |
| Chad | 12.3 (9.3 to 16.6) | 450.48 (343.35 to 613.27) | 29.2 (22.3 to 39.2) | 404.62 (308.89 to 542.11) | 1.38% (1.09 to 1.76) | -0.31% (-0.34 to -0.29) |
| Chile | 31.7 (23.4 to 42.4) | 419.39 (312.14 to 561.49) | 28.1 (20.6 to 38) | 299.07 (220.13 to 402.12) | -0.11% (-0.25 to 0.07) | -1.04% (-1.24 to -0.83) |
| China | 2472.2 (1838.6 to 3256) | 356.04 (261.83 to 470.8) | 1414.4 (1073.1 to 1905.3) | 192 (145.74 to 253.05) | -0.43% (-0.51 to -0.34) | -0.82% (-1.31 to -0.32) |
| Colombia | 39.7 (29.9 to 52.7) | 222.98 (169.33 to 293.88) | 34.6 (26 to 45.7) | 136.51 (102.93 to 179.98) | -0.13% (-0.21 to -0.02) | -1.81% (-1.86 to -1.76) |
| Comoros | 0.8 (0.6 to 1.1) | 399.25 (299.99 to 542.31) | 0.7 (0.5 to 0.9) | 180.84 (133.42 to 245.21) | -0.17% (-0.26 to -0.07) | -2.76% (-2.86 to -2.67) |
| Congo | 3.9 (2.9 to 5.3) | 350.01 (266.02 to 463.16) | 5.7 (4.3 to 7.8) | 207.3 (154.54 to 280.95) | 0.47% (0.29 to 0.65) | -1.44% (-1.6 to -1.28) |
| Cook Islands | 0 (0 to 0) | 301.56 (225.91 to 410.76) | 0 (0 to 0) | 198.32 (143.09 to 267.83) | -0.39% (-0.47 to -0.32) | -1.46% (-1.47 to -1.44) |
| Costa Rica | 3.4 (2.5 to 4.5) | 206.29 (156.5 to 276.2) | 3 (2.2 to 3.9) | 110.9 (81.96 to 146.38) | -0.12% (-0.23 to -0.03) | -2.05% (-2.33 to -1.77) |
| Côte d'Ivoire | 22.1 (16.5 to 29.4) | 402.55 (303.36 to 537.99) | 34.3 (26.1 to 47) | 262.24 (200.22 to 356.48) | 0.55% (0.38 to 0.76) | -1.44% (-1.46 to -1.41) |
| Croatia | 3 (2.2 to 4.1) | 130.88 (92.82 to 178.38) | 2.2 (1.7 to 3) | 123.21 (90.72 to 166.75) | -0.26% (-0.41 to -0.04) | -0.1% (-0.36 to 0.15) |
| Cuba | 5.8 (4.2 to 8) | 89.22 (65.96 to 122.64) | 4.1 (2.9 to 5.6) | 84.69 (61.43 to 115.31) | -0.3% (-0.4 to -0.17) | 0.3% (0.09 to 0.51) |
| Cyprus | 0.9 (0.7 to 1.2) | 225.74 (161.77 to 300.64) | 0.7 (0.5 to 1) | 93.26 (66.46 to 129.73) | -0.19% (-0.34 to 0.05) | -2.97% (-3.34 to -2.6) |
| Czechia | 9.2 (6.7 to 12.5) | 205.42 (147.15 to 278.13) | 8.9 (6.4 to 12.2) | 200.87 (144.34 to 274) | -0.03% (-0.27 to 0.28) | 0.89% (0.35 to 1.44) |
| Democratic People's Republic of Korea | 60.8 (44.1 to 82.9) | 550.77 (399.49 to 752.14) | 27.5 (19.5 to 37.4) | 208.26 (148.69 to 282.71) | -0.55% (-0.63 to -0.47) | -3.26% (-3.34 to -3.18) |
| Democratic Republic of the Congo | 74.2 (56.4 to 101.4) | 441.34 (335.14 to 591.71) | 116.5 (86.6 to 158.5) | 286.07 (212.76 to 391.21) | 0.57% (0.38 to 0.8) | -1.31% (-1.53 to -1.08) |
| Denmark | 5.8 (4.1 to 7.9) | 231.27 (163.95 to 311.25) | 6.3 (4.5 to 8.6) | 257.02 (183.73 to 351.69) | 0.08% (-0.13 to 0.33) | 0.29% (0.25 to 0.34) |
| Djibouti | 0.9 (0.7 to 1.2) | 423.33 (321.59 to 563.75) | 1.5 (1.1 to 2) | 229.24 (171.58 to 308.87) | 0.71% (0.48 to 0.93) | -2.16% (-2.35 to -1.98) |
| Dominica | 0.1 (0 to 0.1) | 173.44 (128.76 to 235.86) | 0 (0 to 0) | 106.08 (78.23 to 145.04) | -0.42% (-0.49 to -0.34) | -1.89% (-1.96 to -1.82) |
| Dominican Republic | 8.5 (6.3 to 11.4) | 212.4 (159.41 to 282.66) | 8.2 (6 to 11.2) | 138.77 (102.57 to 188.37) | -0.04% (-0.15 to 0.11) | -1.54% (-1.72 to -1.36) |
| Ecuador | 16.9 (12.9 to 22.4) | 333.18 (254.04 to 436.06) | 24.6 (19 to 31.3) | 263.74 (203.64 to 334.92) | 0.45% (0.28 to 0.7) | -0.31% (-0.52 to -0.1) |
| Egypt | 65.8 (48.5 to 88.3) | 246.68 (183.14 to 328.54) | 69 (50.9 to 93.6) | 132.02 (97.22 to 178.39) | 0.05% (-0.11 to 0.19) | -2.12% (-2.75 to -1.49) |
| El Salvador | 6.6 (5 to 8.8) | 254.28 (193.1 to 334.16) | 4.8 (3.6 to 6.5) | 136.77 (103.14 to 183.3) | -0.27% (-0.36 to -0.18) | -2.27% (-2.56 to -1.99) |
| Equatorial Guinea | 0.9 (0.7 to 1.2) | 458.95 (349.96 to 607.65) | 1.4 (1.1 to 1.9) | 198.71 (150.9 to 267.97) | 0.54% (0.38 to 0.73) | -3.01% (-3.22 to -2.81) |
| Eritrea | 5.4 (4.1 to 7.2) | 406.53 (307.34 to 544.08) | 8 (6 to 10.7) | 242.9 (183.25 to 326.37) | 0.48% (0.32 to 0.66) | -1.83% (-1.97 to -1.69) |
| Estonia | 2.4 (1.8 to 3.4) | 332.09 (238.8 to 457.27) | 2.1 (1.4 to 2.8) | 341.32 (241.71 to 467.77) | -0.16% (-0.36 to 0.11) | 1.21% (0.81 to 1.62) |
| Eswatini | 1.1 (0.8 to 1.4) | 264.2 (200.35 to 357.34) | 0.9 (0.7 to 1.3) | 142.52 (108.26 to 193.51) | -0.11% (-0.2 to -0.02) | -2.09% (-2.12 to -2.07) |
| Ethiopia | 104.9 (80 to 139.4) | 461.84 (351.52 to 612.17) | 152.1 (114.5 to 203.6) | 292.43 (219.94 to 392.28) | 0.45% (0.35 to 0.55) | -1.67% (-1.77 to -1.58) |
| Fiji | 1 (0.7 to 1.3) | 240.03 (178.08 to 319.95) | 1 (0.7 to 1.4) | 215.51 (154.16 to 294.35) | 0.02% (-0.11 to 0.14) | -0.28% (-0.34 to -0.22) |
| Finland | 5.3 (3.9 to 7.3) | 211.13 (155.19 to 289.69) | 4.3 (3.1 to 6) | 185.06 (133.65 to 254.93) | -0.18% (-0.3 to 0) | -0.55% (-0.74 to -0.37) |
| France | 56.5 (40.6 to 78.2) | 192.31 (137.57 to 267.59) | 59.5 (42.8 to 82.3) | 214.34 (155.02 to 295.28) | 0.05% (-0.11 to 0.27) | 0.57% (0.42 to 0.73) |
| Gabon | 1.5 (1.1 to 2) | 342.65 (260.17 to 453.41) | 1.6 (1.2 to 2.2) | 162.71 (121.88 to 219.6) | 0.06% (-0.07 to 0.17) | -2.52% (-2.56 to -2.49) |
| Gambia | 1.9 (1.5 to 2.6) | 423.5 (326.11 to 569.98) | 2.8 (2.1 to 3.8) | 247.2 (186.49 to 334.03) | 0.47% (0.33 to 0.65) | -1.87% (-2.01 to -1.73) |
| Georgia | 4.6 (3.4 to 6.2) | 161.87 (119.72 to 214.98) | 3 (2.3 to 3.9) | 181.17 (140.61 to 234.63) | -0.36% (-0.48 to -0.19) | 1.24% (0.87 to 1.6) |
| Germany | 29.3 (21 to 40.9) | 71.08 (51.14 to 98.77) | 28.4 (25.2 to 32.4) | 79.78 (70.81 to 91.09) | -0.03% (-0.27 to 0.29) | 0.88% (0.71 to 1.05) |
| Ghana | 23.6 (18 to 31.6) | 340.52 (259.7 to 451.78) | 33.3 (25 to 45.8) | 190.11 (142.67 to 260.54) | 0.41% (0.27 to 0.62) | -1.87% (-2 to -1.74) |
| Greece | 7.3 (5.3 to 9.8) | 146.03 (106.87 to 196.32) | 7.5 (5.3 to 10.4) | 168.05 (117.86 to 232.97) | 0.03% (-0.18 to 0.34) | 0.87% (0.71 to 1.02) |
| Greenland | 0 (0 to 0.1) | 145.13 (108.32 to 196.59) | 0 (0 to 0) | 123.93 (91.62 to 167.53) | -0.31% (-0.39 to -0.2) | -0.72% (-0.83 to -0.62) |
| Grenada | 0.1 (0.1 to 0.1) | 204.49 (153.55 to 281.44) | 0.1 (0 to 0.1) | 120.95 (90.66 to 162.26) | -0.25% (-0.33 to -0.13) | -1.58% (-1.84 to -1.32) |
| Guam | 0.2 (0.1 to 0.3) | 246.11 (185.72 to 334.85) | 0.2 (0.1 to 0.2) | 244.15 (178.11 to 327.43) | -0.02% (-0.14 to 0.14) | -0.18% (-0.37 to 0.01) |
| Guatemala | 16 (12.3 to 21.3) | 464.48 (353.26 to 615.38) | 18.3 (13.9 to 24.3) | 180.94 (136.51 to 239.17) | 0.14% (0.03 to 0.28) | -3.57% (-3.8 to -3.34) |
| Guinea | 12 (9.1 to 16.3) | 418.85 (316.67 to 563.9) | 17.9 (13.7 to 24.1) | 281.39 (214.17 to 380.24) | 0.49% (0.32 to 0.72) | -1.45% (-1.48 to -1.41) |
| Guinea-Bissau | 1.9 (1.4 to 2.5) | 404.11 (306.83 to 542.26) | 2.5 (1.9 to 3.4) | 258.25 (194.79 to 345.67) | 0.35% (0.2 to 0.5) | -1.58% (-1.68 to -1.47) |
| Guyana | 0.8 (0.6 to 1) | 174.42 (130.53 to 231.87) | 0.5 (0.4 to 0.7) | 121.92 (91.11 to 163.17) | -0.32% (-0.41 to -0.22) | -1.09% (-1.15 to -1.02) |
| Haiti | 11.5 (8.6 to 15.2) | 385.83 (287.61 to 510.36) | 15.7 (11.4 to 21.5) | 221.78 (162.21 to 301.98) | 0.36% (0.21 to 0.52) | -1.94% (-1.95 to -1.92) |
| Honduras | 8 (6.2 to 10.6) | 395.65 (300.03 to 517.97) | 9.7 (7.3 to 13) | 176.43 (133.29 to 236.91) | 0.21% (0.09 to 0.35) | -3.07% (-3.27 to -2.86) |
| Hungary | 7 (5 to 9.5) | 156.05 (112.72 to 215.47) | 5.2 (3.8 to 6.9) | 126.94 (91.82 to 170.89) | -0.25% (-0.39 to -0.06) | -0.3% (-0.62 to 0.03) |
| Iceland | 0.3 (0.2 to 0.5) | 247.68 (180.29 to 334.56) | 0.3 (0.2 to 0.4) | 195.34 (141.94 to 271.23) | -0.07% (-0.24 to 0.1) | -0.53% (-0.75 to -0.31) |
| India | 1156.3 (878.6 to 1537.3) | 273.63 (209.16 to 361.83) | 930.8 (681.7 to 1260.7) | 122.25 (89.78 to 165.19) | -0.2% (-0.26 to -0.1) | -2.85% (-2.9 to -2.79) |
| Indonesia | 133.4 (99.3 to 180.3) | 135.93 (102.13 to 183.69) | 119.5 (90.1 to 162.1) | 85.6 (64.6 to 116.1) | -0.1% (-0.2 to 0) | -1.37% (-1.5 to -1.24) |
| Iran (Islamic Republic of) | 31.8 (24.2 to 42.6) | 123.53 (93.78 to 170.1) | 26.8 (19.7 to 38.4) | 52.5 (38.9 to 73.41) | -0.16% (-0.26 to -0.06) | -2.38% (-2.82 to -1.94) |
| Iraq | 24 (18.3 to 31.5) | 326.2 (245.44 to 430.9) | 31.2 (23.3 to 42.2) | 138.48 (102.96 to 187.46) | 0.3% (0.13 to 0.44) | -3.11% (-3.19 to -3.03) |
| Ireland | 4.3 (3.1 to 5.9) | 247.66 (179.88 to 336.92) | 5.5 (3.9 to 7.5) | 221.95 (163.4 to 303.01) | 0.26% (0.01 to 0.51) | -0.01% (-0.16 to 0.14) |
| Israel | 7.9 (5.8 to 10.8) | 320.39 (234.85 to 439.27) | 15.7 (11.3 to 21.4) | 366.15 (263.76 to 501.65) | 0.99% (0.73 to 1.3) | 0.39% (0.29 to 0.5) |
| Italy | 26.3 (16.9 to 39.3) | 92.35 (59.5 to 137.76) | 35.1 (25.9 to 46.3) | 144.47 (106.46 to 190.36) | 0.33% (0.02 to 0.7) | 1.61% (1.21 to 2) |
| Jamaica | 2 (1.5 to 2.7) | 155.93 (116.34 to 212.52) | 1.5 (1.1 to 2.1) | 95.2 (70.67 to 129.92) | -0.24% (-0.35 to -0.11) | -1.76% (-1.84 to -1.68) |
| Japan | 41.2 (29.4 to 57.6) | 74.04 (52.68 to 104.03) | 32.5 (23.6 to 45.5) | 69.28 (50.29 to 96.12) | -0.21% (-0.31 to -0.08) | -0.04% (-0.21 to 0.14) |
| Jordan | 5.7 (4.2 to 7.6) | 368.62 (267.35 to 499.25) | 12 (9 to 15.7) | 209.41 (156.8 to 276.8) | 1.12% (0.87 to 1.45) | -1.69% (-1.87 to -1.5) |
| Kazakhstan | 22.2 (16.6 to 29.7) | 249.83 (187.3 to 333.55) | 23.2 (17 to 31.5) | 237.25 (173.67 to 320.64) | 0.05% (-0.08 to 0.23) | 0.78% (0.34 to 1.22) |
| Kenya | 41.7 (32 to 56.4) | 400.14 (307.34 to 530.61) | 50.8 (38.5 to 68.6) | 183.49 (138.53 to 247.39) | 0.22% (0.17 to 0.27) | -2.6% (-2.64 to -2.55) |
| Kiribati | 0.2 (0.1 to 0.2) | 395.59 (291.69 to 536.97) | 0.2 (0.1 to 0.3) | 288.23 (210.45 to 392.65) | 0.22% (0.09 to 0.38) | -1.03% (-1.11 to -0.96) |
| Kuwait | 1 (0.7 to 1.3) | 106.88 (80.83 to 140.77) | 1.5 (1.1 to 2.1) | 47.86 (35.12 to 64.91) | 0.58% (0.41 to 0.76) | -3.89% (-4.73 to -3.04) |
| Kyrgyzstan | 7.6 (5.6 to 10.2) | 333.67 (246.79 to 443.55) | 9.1 (6.7 to 12.3) | 255.61 (188.64 to 342.89) | 0.2% (0.04 to 0.37) | 0.06% (-0.37 to 0.5) |
| Lao People's Democratic Republic | 5.3 (4 to 7) | 281.53 (211.71 to 368.33) | 5.3 (3.9 to 7) | 130.68 (96.91 to 175.12) | -0.01% (-0.15 to 0.13) | -3.06% (-3.24 to -2.89) |
| Latvia | 4.6 (3.3 to 6.2) | 363.81 (264.04 to 494.92) | 3.2 (2.3 to 4.4) | 383.05 (276 to 519.24) | -0.3% (-0.46 to -0.1) | 1.06% (0.51 to 1.62) |
| Lebanon | 3.5 (2.6 to 4.6) | 225.11 (169.41 to 299.43) | 3 (2.2 to 4) | 104.15 (77.65 to 139.03) | -0.15% (-0.25 to -0.04) | -2.55% (-2.62 to -2.49) |
| Lesotho | 2.1 (1.6 to 2.9) | 248.63 (190.38 to 334.51) | 1.6 (1.2 to 2.1) | 132.67 (99.59 to 177.64) | -0.27% (-0.36 to -0.19) | -2.02% (-2.12 to -1.91) |
| Liberia | 3.5 (2.7 to 4.8) | 402.36 (304.99 to 545.19) | 5.1 (3.8 to 7) | 204.33 (153.33 to 276.51) | 0.46% (0.31 to 0.6) | -2.33% (-2.5 to -2.16) |
| Libya | 4.1 (3.1 to 5.5) | 259.92 (195.62 to 351.88) | 3.8 (2.7 to 5.1) | 91.62 (65.95 to 123.97) | -0.07% (-0.21 to 0.09) | -3.18% (-3.38 to -2.98) |
| Lithuania | 5.8 (4.2 to 7.8) | 311.97 (227.23 to 416.35) | 3.6 (2.6 to 5) | 302.26 (217.13 to 420.77) | -0.37% (-0.49 to -0.22) | 0.56% (0.03 to 1.1) |
| Luxembourg | 0.4 (0.3 to 0.6) | 211.81 (148.7 to 284.01) | 0.7 (0.5 to 0.9) | 214.08 (150.48 to 289.47) | 0.56% (0.27 to 0.96) | -0.13% (-0.23 to -0.02) |
| Madagascar | 20.4 (15.1 to 27.4) | 378.23 (281.38 to 510.19) | 31.8 (24 to 43.7) | 231.66 (175.44 to 316.13) | 0.56% (0.4 to 0.75) | -1.69% (-1.79 to -1.6) |
| Malawi | 18.8 (14.2 to 25.2) | 430.36 (327.57 to 569.63) | 22.3 (16.4 to 30) | 238.2 (180.31 to 316.19) | 0.19% (0.06 to 0.31) | -2.12% (-2.33 to -1.92) |
| Malaysia | 17.2 (12.7 to 23.4) | 186.95 (137.9 to 252.85) | 20.1 (14.6 to 27.8) | 115.71 (83.81 to 159.22) | 0.17% (0.03 to 0.32) | -2.01% (-2.18 to -1.84) |
| Maldives | 0.2 (0.2 to 0.3) | 251.97 (187.81 to 336.75) | 0.3 (0.2 to 0.4) | 104.72 (76.79 to 142.42) | 0.19% (0.04 to 0.38) | -2.69% (-3.07 to -2.3) |
| Mali | 17.8 (13.5 to 23.8) | 463.49 (352.53 to 611.33) | 36.4 (27.6 to 49) | 367.74 (280.7 to 487.36) | 1.04% (0.8 to 1.29) | -0.85% (-0.89 to -0.81) |
| Malta | 0.2 (0.1 to 0.3) | 102.3 (73.75 to 141.21) | 0.2 (0.1 to 0.2) | 77.95 (55.93 to 108.67) | -0.18% (-0.32 to -0.05) | -1.01% (-1.52 to -0.49) |
| Marshall Islands | 0.1 (0.1 to 0.1) | 365.57 (270.12 to 486.59) | 0.1 (0.1 to 0.1) | 229.38 (170.14 to 310.22) | -0.09% (-0.2 to 0.04) | -1.36% (-1.47 to -1.25) |
| Mauritania | 3.7 (2.8 to 4.9) | 395.66 (298.34 to 534.67) | 4.4 (3.3 to 6) | 226.39 (168.16 to 306.96) | 0.21% (0.08 to 0.37) | -1.73% (-1.88 to -1.59) |
| Mauritius | 0.7 (0.5 to 0.9) | 101.03 (74.71 to 136.27) | 0.4 (0.3 to 0.6) | 67.2 (49.21 to 91.37) | -0.36% (-0.44 to -0.26) | -1.55% (-1.63 to -1.47) |
| Mexico | 88.9 (63 to 124.5) | 202.86 (142.75 to 287.02) | 106.9 (83.5 to 139.1) | 158.21 (123.36 to 205.48) | 0.2% (0.04 to 0.43) | -1.75% (-2.04 to -1.46) |
| Micronesia (Federated States of) | 0.2 (0.2 to 0.3) | 508 (377.72 to 679.62) | 0.1 (0.1 to 0.2) | 236.53 (169.84 to 319.75) | -0.47% (-0.54 to -0.39) | -2.6% (-2.69 to -2.5) |
| Monaco | 0 (0 to 0) | 183.72 (134.74 to 251.09) | 0 (0 to 0) | 177.78 (126.2 to 248) | -0.03% (-0.17 to 0.17) | -0.11% (-0.15 to -0.07) |
| Mongolia | 4.6 (3.4 to 6.1) | 428.03 (321.89 to 564.34) | 5.7 (4.2 to 7.8) | 302.95 (224.76 to 408.75) | 0.25% (0.06 to 0.5) | -0.27% (-0.91 to 0.38) |
| Montenegro | 0.5 (0.4 to 0.7) | 172.28 (123.05 to 233.48) | 0.4 (0.3 to 0.6) | 143.75 (102.05 to 194.85) | -0.26% (-0.37 to -0.15) | -0.66% (-0.72 to -0.59) |
| Morocco | 28 (20.8 to 37.9) | 230.5 (170.77 to 312.49) | 23.8 (17.5 to 32.1) | 121.45 (89.87 to 163.72) | -0.15% (-0.24 to -0.04) | -2.19% (-2.25 to -2.14) |
| Mozambique | 23.6 (18.3 to 31.5) | 384.75 (296.72 to 507.82) | 41.9 (31.7 to 55.6) | 293.09 (223.67 to 388.65) | 0.77% (0.57 to 0.96) | -0.85% (-0.98 to -0.71) |
| Myanmar | 43.4 (33.1 to 57.3) | 213.91 (162.03 to 281.07) | 35.9 (26.8 to 48.3) | 119.58 (89.14 to 160.59) | -0.17% (-0.26 to -0.06) | -1.93% (-1.97 to -1.88) |
| Namibia | 1.7 (1.2 to 2.2) | 254.53 (192.3 to 339.6) | 2.1 (1.6 to 2.8) | 156.72 (117.56 to 211.87) | 0.25% (0.08 to 0.41) | -1.51% (-1.6 to -1.42) |
| Nauru | 0 (0 to 0) | 500.75 (364.69 to 684.33) | 0 (0 to 0) | 289.32 (213.53 to 390.31) | -0.34% (-0.4 to -0.26) | -1.98% (-2.02 to -1.94) |
| Nepal | 33.9 (25.2 to 44.7) | 352.32 (266.18 to 466.57) | 22.9 (16.1 to 31.1) | 119.29 (84.86 to 161.97) | -0.33% (-0.42 to -0.22) | -4.12% (-4.3 to -3.94) |
| Netherlands | 15 (10.5 to 20.8) | 183.62 (128.97 to 255.2) | 14.4 (9.9 to 20) | 198.49 (137.46 to 275.93) | -0.04% (-0.18 to 0.18) | 0.43% (0.3 to 0.56) |
| New Zealand | 4.4 (2.7 to 6.9) | 235.43 (147.46 to 369.72) | 5.5 (4.2 to 7.2) | 281.82 (214.4 to 369.58) | 0.26% (-0.07 to 0.82) | -0.12% (-0.34 to 0.09) |
| Nicaragua | 6 (4.5 to 8) | 326.69 (246.26 to 438.56) | 5.4 (4.1 to 7.2) | 148.32 (112.64 to 195.45) | -0.09% (-0.18 to 0.03) | -2.57% (-2.93 to -2.21) |
| Niger | 17 (13 to 22.8) | 485 (373.73 to 645.85) | 42.5 (31.9 to 57) | 444.61 (334.52 to 595.95) | 1.49% (1.19 to 1.77) | -0.3% (-0.37 to -0.24) |
| Nigeria | 181.4 (139.7 to 239.9) | 463.29 (357.87 to 612.11) | 328.8 (253.1 to 436.7) | 318.31 (242.43 to 421.56) | 0.81% (0.74 to 0.89) | -1.25% (-1.38 to -1.13) |
| Niue | 0 (0 to 0) | 348.18 (260.99 to 476.69) | 0 (0 to 0) | 218.41 (158.94 to 290.44) | -0.53% (-0.58 to -0.46) | -1.65% (-1.68 to -1.61) |
| North Macedonia | 1.9 (1.4 to 2.6) | 182.49 (131.91 to 250.93) | 1.2 (0.9 to 1.7) | 119.68 (84.82 to 163.42) | -0.34% (-0.46 to -0.22) | -1.48% (-1.71 to -1.25) |
| Northern Mariana Islands | 0.1 (0 to 0.1) | 210.4 (156.19 to 283.24) | 0 (0 to 0) | 176.63 (128.74 to 238.38) | -0.58% (-0.64 to -0.52) | -0.39% (-0.46 to -0.33) |
| Norway | 3.5 (2.2 to 5.3) | 164.65 (102.41 to 250.36) | 3.7 (2.3 to 5.9) | 151.74 (92.47 to 236.77) | 0.07% (-0.1 to 0.28) | -0.08% (-0.24 to 0.08) |
| Oman | 2.5 (1.9 to 3.5) | 374.78 (278.83 to 509.26) | 2.6 (1.8 to 3.5) | 116.18 (84.11 to 159.42) | 0.02% (-0.11 to 0.18) | -4.27% (-4.46 to -4.08) |
| Pakistan | 221.7 (169.4 to 296.3) | 463.99 (354.51 to 614.65) | 292.2 (219.9 to 391.1) | 253.31 (191.22 to 335.39) | 0.32% (0.2 to 0.44) | -2.11% (-2.26 to -1.96) |
| Palau | 0 (0 to 0) | 241.86 (180.56 to 322.11) | 0 (0 to 0) | 169.84 (123.39 to 231.3) | -0.41% (-0.48 to -0.32) | -1.2% (-1.25 to -1.16) |
| Palestine | 2.9 (2.2 to 3.9) | 337.72 (249.64 to 460.96) | 3.5 (2.6 to 4.6) | 131.98 (99.05 to 177.51) | 0.19% (0.04 to 0.34) | -3.42% (-3.67 to -3.17) |
| Panama | 2.3 (1.7 to 3.2) | 183.62 (136.85 to 245.65) | 3.3 (2.4 to 4.3) | 157.61 (117.83 to 209.54) | 0.4% (0.23 to 0.6) | -0.45% (-0.59 to -0.31) |
| Papua New Guinea | 8.9 (6.6 to 11.9) | 476.02 (352 to 630.99) | 20.5 (15.3 to 27.4) | 407.76 (303.16 to 544.52) | 1.31% (1.06 to 1.62) | -0.58% (-0.6 to -0.55) |
| Paraguay | 3 (2.3 to 4.1) | 160.05 (121.59 to 217.35) | 3.1 (2.3 to 4.3) | 81.37 (59.54 to 111.57) | 0.02% (-0.09 to 0.14) | -2.29% (-2.33 to -2.24) |
| Peru | 42.8 (32.3 to 57) | 399.6 (302.8 to 531.27) | 43.1 (31.7 to 58.4) | 237.41 (175.54 to 320.6) | 0.01% (-0.12 to 0.15) | -1.74% (-1.83 to -1.64) |
| Philippines | 92.7 (70 to 123.4) | 302.25 (228.31 to 403.5) | 112.3 (84.7 to 150.6) | 194.36 (146.89 to 260.19) | 0.21% (0.16 to 0.26) | -1.51% (-1.58 to -1.45) |
| Poland | 11.4 (8.2 to 15.9) | 64.05 (45.43 to 89.95) | 8.4 (7.1 to 10.1) | 47.27 (39.96 to 56.41) | -0.26% (-0.42 to -0.04) | -0.8% (-1.24 to -0.36) |
| Portugal | 5.3 (3.9 to 7.4) | 107.29 (77.46 to 149.04) | 4.3 (3.2 to 5.8) | 95.04 (71.33 to 127.18) | -0.19% (-0.35 to 0.01) | -0.32% (-0.4 to -0.24) |
| Puerto Rico | 2.3 (1.7 to 3.1) | 118.47 (86.56 to 160.37) | 1 (0.7 to 1.3) | 62.54 (46.06 to 84.6) | -0.57% (-0.63 to -0.51) | -2.35% (-2.57 to -2.13) |
| Qatar | 0.4 (0.3 to 0.6) | 236.38 (172.96 to 318.11) | 1.2 (0.9 to 1.7) | 108.33 (78.62 to 147.82) | 1.95% (1.58 to 2.41) | -2.78% (-2.84 to -2.72) |
| Republic of Korea | 21.2 (14.9 to 29.9) | 75.21 (52.78 to 105.62) | 11.5 (9.9 to 13.4) | 47.95 (41.11 to 55.93) | -0.46% (-0.59 to -0.25) | -2.27% (-2.6 to -1.93) |
| Republic of Moldova | 9 (6.5 to 12) | 390.79 (285.32 to 519.43) | 4.2 (3 to 5.6) | 232.81 (167.52 to 306.78) | -0.53% (-0.61 to -0.42) | -1.25% (-1.57 to -0.94) |
| Romania | 20.5 (14.8 to 27) | 184.66 (132.95 to 245.97) | 13.9 (10.4 to 18.6) | 186.61 (136.44 to 248.52) | -0.32% (-0.46 to -0.15) | 0.54% (0.2 to 0.89) |
| Russian Federation | 243.4 (184.4 to 319.7) | 329.08 (250.91 to 429.2) | 261.9 (195.1 to 350.6) | 372.05 (281.06 to 485.62) | 0.08% (-0.09 to 0.26) | 1.74% (1.13 to 2.36) |
| Rwanda | 12.6 (9.4 to 17) | 412.57 (311.36 to 551.44) | 13.9 (10.3 to 18.9) | 212.63 (157.2 to 285.51) | 0.1% (-0.09 to 0.25) | -2.59% (-2.74 to -2.44) |
| Saint Kitts and Nevis | 0 (0 to 0) | 150.73 (112.95 to 200.94) | 0 (0 to 0) | 90.96 (67.83 to 123.14) | -0.14% (-0.24 to -0.01) | -1.77% (-1.8 to -1.74) |
| Saint Lucia | 0.1 (0.1 to 0.2) | 173.4 (129.53 to 233.99) | 0.1 (0.1 to 0.1) | 84.32 (62.61 to 113.79) | -0.38% (-0.46 to -0.27) | -2.51% (-2.67 to -2.36) |
| Saint Vincent and the Grenadines | 0.1 (0.1 to 0.1) | 165.44 (122.83 to 223.27) | 0.1 (0 to 0.1) | 111.92 (82.12 to 152.04) | -0.33% (-0.43 to -0.24) | -1.33% (-1.47 to -1.2) |
| Samoa | 0.2 (0.1 to 0.2) | 231.06 (168.08 to 313.35) | 0.1 (0.1 to 0.2) | 120.26 (86.34 to 164.29) | -0.3% (-0.39 to -0.21) | -2.25% (-2.35 to -2.15) |
| San Marino | 0 (0 to 0) | 172.54 (124.5 to 238.61) | 0 (0 to 0) | 177.6 (127.79 to 241.08) | 0.33% (0.1 to 0.63) | 0.1% (0.08 to 0.12) |
| Sao Tome and Principe | 0.2 (0.1 to 0.2) | 364.95 (272.67 to 487.64) | 0.2 (0.1 to 0.2) | 174.22 (131.08 to 232.06) | 0.01% (-0.13 to 0.17) | -2.61% (-2.78 to -2.44) |
| Saudi Arabia | 19.1 (14 to 25.7) | 307.3 (228.8 to 408.77) | 24 (17.6 to 33.3) | 109.23 (80.63 to 151.21) | 0.26% (0.08 to 0.46) | -3.68% (-3.73 to -3.63) |
| Senegal | 13.8 (10.4 to 18.5) | 411.79 (308.69 to 553.99) | 18.3 (13.9 to 25) | 251.06 (187.98 to 342.3) | 0.33% (0.17 to 0.49) | -1.47% (-1.59 to -1.35) |
| Serbia | 7.5 (5.4 to 10.3) | 173.46 (124.06 to 235.01) | 4.6 (3.3 to 6.2) | 120.33 (87.24 to 162.23) | -0.39% (-0.5 to -0.26) | -1.33% (-1.7 to -0.95) |
| Seychelles | 0 (0 to 0.1) | 121.59 (90.95 to 163.14) | 0 (0 to 0.1) | 104.04 (77.14 to 141.36) | 0.05% (-0.1 to 0.21) | -0.16% (-0.43 to 0.12) |
| Sierra Leone | 6.3 (4.8 to 8.5) | 359.04 (277.07 to 484.14) | 10.4 (7.9 to 14.2) | 241.83 (183.44 to 321.7) | 0.66% (0.46 to 0.86) | -1.4% (-1.55 to -1.24) |
| Singapore | 1.5 (1.1 to 2.1) | 74.65 (53.78 to 104.21) | 1.8 (1.3 to 2.6) | 53 (37.72 to 74.38) | 0.18% (0 to 0.39) | -1.34% (-1.78 to -0.89) |
| Slovakia | 5.5 (4 to 7.4) | 220.3 (158.26 to 294.67) | 4.3 (3.1 to 5.7) | 170.71 (126.28 to 226.21) | -0.23% (-0.39 to 0.01) | -0.42% (-1.08 to 0.25) |
| Slovenia | 1.2 (0.9 to 1.6) | 123.38 (90.7 to 167.07) | 1.2 (0.8 to 1.7) | 141.76 (100.09 to 196.13) | -0.02% (-0.23 to 0.22) | 1.18% (0.9 to 1.46) |
| Solomon Islands | 0.8 (0.6 to 1.1) | 615.5 (455.35 to 809.18) | 1.3 (0.9 to 1.7) | 393.91 (289.65 to 534.16) | 0.5% (0.35 to 0.7) | -1.58% (-1.67 to -1.49) |
| Somalia | 13.9 (10.7 to 18.5) | 462.72 (355.09 to 612.37) | 35.3 (26.4 to 46.8) | 403.13 (302.3 to 532.44) | 1.53% (1.25 to 2) | -0.49% (-0.54 to -0.44) |
| South Africa | 14.5 (11.1 to 18.9) | 71.73 (54.78 to 93.56) | 12.8 (9.8 to 16.9) | 40.59 (31.34 to 53.1) | -0.12% (-0.2 to -0.04) | -2.16% (-2.32 to -2) |
| South Sudan | 9.6 (7.2 to 12.7) | 376.02 (287.11 to 490.52) | 14.4 (10.9 to 19.2) | 333.72 (252.64 to 450.46) | 0.5% (0.33 to 0.72) | -0.4% (-0.45 to -0.34) |
| Spain | 29.8 (21.5 to 41.1) | 153.87 (111.99 to 212.67) | 32.9 (23.7 to 46) | 157.58 (114.02 to 214.03) | 0.11% (-0.12 to 0.43) | 0.48% (0.33 to 0.63) |
| Sri Lanka | 11.8 (8.6 to 15.9) | 123.93 (91.14 to 167.71) | 10.7 (7.7 to 14.9) | 96.66 (69.7 to 133.56) | -0.09% (-0.2 to 0.04) | -0.54% (-0.72 to -0.36) |
| Sudan | 33.5 (25.5 to 45.8) | 360 (270.32 to 489.97) | 38 (28.9 to 50.9) | 176.67 (133.76 to 237.59) | 0.13% (-0.01 to 0.27) | -2.53% (-2.69 to -2.36) |
| Suriname | 0.3 (0.3 to 0.5) | 172.84 (126.68 to 233.69) | 0.4 (0.3 to 0.5) | 131.58 (96.37 to 178.31) | 0.08% (-0.07 to 0.27) | -0.83% (-0.96 to -0.7) |
| Sweden | 16.5 (12.2 to 21.9) | 421.21 (310.54 to 558.6) | 15 (10.8 to 20.4) | 331.43 (241.33 to 450.32) | -0.09% (-0.24 to 0.11) | -0.47% (-0.77 to -0.17) |
| Switzerland | 7.7 (5.5 to 10.7) | 210.93 (149.94 to 291.04) | 9 (6.4 to 12.3) | 215.39 (153.85 to 292.13) | 0.17% (-0.07 to 0.49) | 0.11% (-0.07 to 0.3) |
| Syrian Arab Republic | 14.7 (11.2 to 19.3) | 273.17 (205.61 to 361.55) | 7.3 (5.4 to 9.7) | 102.69 (76.1 to 138.67) | -0.5% (-0.56 to -0.43) | -2.87% (-3.38 to -2.35) |
| Taiwan (Province of China) | 24.1 (17.4 to 33.2) | 196.75 (143.3 to 270.46) | 21 (15 to 28.9) | 172.32 (123.61 to 237.45) | -0.13% (-0.33 to 0.14) | -0.84% (-1.31 to -0.37) |
| Tajikistan | 12.8 (9.3 to 16.8) | 501.28 (375.74 to 658.47) | 17.1 (12.4 to 23.2) | 322.1 (237.28 to 434) | 0.34% (0.15 to 0.54) | -1.31% (-1.55 to -1.07) |
| Thailand | 31.4 (23.7 to 42.2) | 93.26 (70.8 to 124.16) | 18.6 (13.8 to 25) | 55.08 (40.75 to 74.08) | -0.41% (-0.49 to -0.32) | -1.62% (-1.74 to -1.51) |
| Timor-Leste | 1.3 (1 to 1.8) | 356.56 (267.59 to 473.14) | 1.3 (1 to 1.8) | 225.01 (167.33 to 304.44) | 0.01% (-0.1 to 0.13) | -1.73% (-1.98 to -1.48) |
| Togo | 6.5 (5 to 8.8) | 392.66 (300.68 to 529.97) | 9.5 (7.1 to 12.9) | 229.56 (171.92 to 311.03) | 0.46% (0.29 to 0.67) | -1.62% (-1.76 to -1.48) |
| Tokelau | 0 (0 to 0) | 557.28 (413.24 to 738.2) | 0 (0 to 0) | 379.01 (275.23 to 518.05) | -0.39% (-0.47 to -0.3) | -1.34% (-1.36 to -1.32) |
| Tonga | 0.2 (0.1 to 0.2) | 390.09 (287.09 to 526.98) | 0.1 (0.1 to 0.2) | 313.29 (227.58 to 422.09) | -0.05% (-0.15 to 0.09) | -0.66% (-0.73 to -0.59) |
| Trinidad and Tobago | 0.9 (0.7 to 1.2) | 136.68 (100.11 to 184.88) | 0.7 (0.5 to 0.9) | 95.62 (70.52 to 129.22) | -0.24% (-0.33 to -0.14) | -0.76% (-1.02 to -0.51) |
| Tunisia | 7.6 (5.7 to 10.2) | 184.78 (139.63 to 248.87) | 6.4 (4.6 to 8.8) | 95.91 (69.62 to 131.68) | -0.16% (-0.29 to -0.02) | -1.92% (-2.11 to -1.73) |
| Turkey | 75.7 (56.9 to 101.3) | 244.04 (182.12 to 322.53) | 67.4 (49.5 to 91.9) | 149.19 (110 to 201.82) | -0.11% (-0.26 to 0.07) | -1.62% (-1.73 to -1.5) |
| Turkmenistan | 8.1 (5.9 to 10.9) | 430.19 (316.91 to 584.46) | 6.7 (4.8 to 9.1) | 262.41 (188.74 to 353.13) | -0.17% (-0.28 to -0.01) | -1.42% (-1.84 to -0.99) |
| Tuvalu | 0 (0 to 0) | 366.3 (267.22 to 485.47) | 0 (0 to 0) | 231.11 (166.98 to 317.05) | -0.3% (-0.39 to -0.2) | -1.62% (-1.65 to -1.59) |
| Uganda | 34.3 (25.7 to 46.6) | 442.56 (335.59 to 595.19) | 57.4 (42.7 to 77.9) | 287.38 (217.29 to 389.03) | 0.67% (0.48 to 0.92) | -1.58% (-1.76 to -1.4) |
| Ukraine | 80.2 (59.6 to 109.2) | 330.24 (245.77 to 452.72) | 60.9 (44.9 to 81.4) | 295.78 (219.25 to 394.48) | -0.24% (-0.41 to -0.06) | 0.75% (0.15 to 1.36) |
| United Arab Emirates | 1.6 (1.2 to 2.1) | 211.8 (158.94 to 281.59) | 2.7 (1.9 to 3.8) | 66.37 (48.57 to 89.29) | 0.75% (0.42 to 1.15) | -3.73% (-3.84 to -3.61) |
| United Kingdom | 61.9 (46.2 to 80.6) | 216.34 (161.94 to 281.19) | 69 (51.1 to 92.4) | 220.28 (164.55 to 291.73) | 0.11% (-0.02 to 0.28) | 0.43% (0.23 to 0.63) |
| United Republic of Tanzania | 46.8 (35.4 to 62.3) | 395.59 (299.88 to 524.84) | 78.9 (59.7 to 106.1) | 277.46 (211.21 to 373.4) | 0.69% (0.5 to 0.89) | -1.09% (-1.18 to -1.01) |
| United States of America | 177.3 (123.7 to 245.2) | 128.89 (90.49 to 178.25) | 114 (97.1 to 139.1) | 76.28 (64.96 to 92.83) | -0.36% (-0.49 to -0.15) | -2.69% (-3.17 to -2.21) |
| United States Virgin Islands | 0.1 (0.1 to 0.1) | 163.72 (121.77 to 221.16) | 0 (0 to 0.1) | 102.78 (75.46 to 139.7) | -0.5% (-0.56 to -0.44) | -1.77% (-1.86 to -1.69) |
| Uruguay | 6 (4.5 to 8) | 401.04 (296.35 to 533.94) | 5.3 (3.9 to 7.2) | 318.99 (237.44 to 434.63) | -0.13% (-0.25 to 0) | -0.93% (-1.08 to -0.78) |
| Uzbekistan | 42.7 (30.9 to 57.8) | 391.34 (285.34 to 522.46) | 41.7 (30.2 to 56.1) | 218.38 (158.65 to 291.91) | -0.02% (-0.16 to 0.17) | -1.77% (-2.15 to -1.39) |
| Vanuatu | 0.3 (0.2 to 0.4) | 467.5 (348.29 to 635.87) | 0.4 (0.3 to 0.6) | 286.33 (211.4 to 380.44) | 0.31% (0.17 to 0.51) | -1.8% (-1.84 to -1.75) |
| Venezuela (Bolivarian Republic of) | 24.8 (18.6 to 33.7) | 247.63 (186.69 to 333.47) | 21 (16 to 28.3) | 143.24 (108.8 to 193.16) | -0.15% (-0.26 to -0.06) | -1.4% (-1.64 to -1.16) |
| Viet Nam | 51.5 (37.5 to 70.4) | 139.5 (103.74 to 188.87) | 41.4 (30.4 to 57.2) | 75.71 (56 to 104.12) | -0.2% (-0.3 to -0.08) | -1.74% (-1.89 to -1.59) |
| Yemen | 23.1 (17.4 to 30.9) | 422.14 (316.26 to 564.14) | 31.9 (24.2 to 42.2) | 202.16 (153.73 to 267.61) | 0.38% (0.24 to 0.54) | -2.64% (-2.83 to -2.44) |
| Zambia | 14.6 (11 to 19.8) | 406.32 (303.09 to 543.83) | 22.6 (16.9 to 30.8) | 239.78 (180.53 to 325.97) | 0.54% (0.37 to 0.71) | -1.78% (-1.94 to -1.62) |
| Zimbabwe | 12.6 (9.5 to 17) | 259.28 (195.48 to 352.53) | 14.5 (10.7 to 20) | 175.33 (131.1 to 239.99) | 0.15% (0.04 to 0.28) | -0.94% (-1.1 to -0.79) |
