# Supplementary material for: Global burden and trends of ectopic pregnancy: An observational trend study from 1990 to 2019
Source: PLoS One. 2023 Oct 26;18(10):e0291316. doi: 10.1371/journal.pone.0291316 (PMC10602312; doi:10.1371/journal.pone.0291316)
Supplement: S2 Table — (DOCX) [file pone.0291316.s002.docx]

S3 Table. Supplementary Table 3. DALYs of ectopic pregnancy in 1990 and 2019 for all locations, with EAPC from 1990 and 2019.

| location | Num_1990 | ASR_1990 | Num_2019 | ASR_2019 | Num_change | EAPC_CI |
| --- | --- | --- | --- | --- | --- | --- |
| Afghanistan | 0.3 (0.2 to 0.4) | 6.68 (4.82 to 9.08) | 0.7 (0.5 to 1) | 4.32 (3.07 to 6.13) | 1.25% (0.57 to 2.32) | -1.28% (-1.71 to -0.85) |
| Albania | 0 (0 to 0) | 1.5 (1.17 to 1.87) | 0 (0 to 0) | 0.6 (0.42 to 0.83) | -0.72% (-0.81 to -0.59) | -1.9% (-2.29 to -1.51) |
| Algeria | 0.4 (0.3 to 0.6) | 3.59 (2.6 to 4.92) | 0.2 (0.2 to 0.3) | 0.93 (0.69 to 1.23) | -0.47% (-0.64 to -0.2) | -4.55% (-4.8 to -4.3) |
| American Samoa | 0 (0 to 0) | 4.23 (3.08 to 5.59) | 0 (0 to 0) | 6.16 (4.3 to 8.61) | 0.64% (-0.01 to 1.58) | 1.09% (0.59 to 1.59) |
| Andorra | 0 (0 to 0) | 0.31 (0.21 to 0.42) | 0 (0 to 0) | 0.2 (0.13 to 0.29) | -0.23% (-0.44 to 0.02) | -1.59% (-1.64 to -1.54) |
| Angola | 2.7 (1.8 to 3.7) | 60.19 (39.58 to 83.16) | 7.5 (4.8 to 10.4) | 54.21 (33.99 to 75.71) | 1.79% (0.65 to 3.37) | 0.09% (-0.2 to 0.37) |
| Antigua and Barbuda | 0 (0 to 0) | 1.84 (1.48 to 2.31) | 0 (0 to 0) | 5.54 (4.3 to 7.07) | 3.09% (1.92 to 4.62) | 4.7% (3.9 to 5.51) |
| Argentina | 0.1 (0.1 to 0.2) | 0.86 (0.59 to 1.22) | 0.4 (0.4 to 0.5) | 1.86 (1.53 to 2.27) | 2.15% (1.3 to 3.42) | 3.04% (2.18 to 3.91) |
| Armenia | 0 (0 to 0) | 0.85 (0.66 to 1.07) | 0 (0 to 0) | 0.34 (0.24 to 0.45) | -0.7% (-0.78 to -0.6) | -2.28% (-3.16 to -1.38) |
| Australia | 0.1 (0.1 to 0.1) | 0.8 (0.63 to 0.99) | 0 (0 to 0) | 0.25 (0.2 to 0.31) | -0.59% (-0.7 to -0.44) | -3.81% (-4.62 to -3) |
| Austria | 0.1 (0 to 0.1) | 1.21 (0.96 to 1.54) | 0 (0 to 0) | 0.42 (0.21 to 0.72) | -0.67% (-0.83 to -0.47) | -4.48% (-5.28 to -3.68) |
| Azerbaijan | 0 (0 to 0) | 0.74 (0.57 to 0.95) | 0 (0 to 0) | 0.3 (0.21 to 0.42) | -0.48% (-0.61 to -0.31) | -3.03% (-3.47 to -2.59) |
| Bahamas | 0 (0 to 0) | 4.65 (3.74 to 5.82) | 0 (0 to 0) | 11.5 (8.2 to 15.74) | 2.29% (1.21 to 3.78) | 3.09% (2.17 to 4.03) |
| Bahrain | 0 (0 to 0) | 1.5 (1.15 to 1.91) | 0 (0 to 0) | 0.5 (0.37 to 0.68) | -0.05% (-0.35 to 0.43) | -2.93% (-3.47 to -2.38) |
| Bangladesh | 0.7 (0.5 to 0.9) | 1.34 (0.98 to 1.71) | 2 (1.4 to 2.6) | 2.12 (1.52 to 2.86) | 1.65% (0.8 to 2.93) | 4.38% (3.24 to 5.54) |
| Barbados | 0 (0 to 0) | 2.96 (2.41 to 3.62) | 0 (0 to 0) | 8.62 (6.4 to 11.41) | 1.71% (0.93 to 2.82) | 4.47% (3.43 to 5.53) |
| Belarus | 0.1 (0.1 to 0.1) | 1.51 (1.21 to 1.9) | 0 (0 to 0) | 0.63 (0.4 to 0.95) | -0.66% (-0.78 to -0.49) | -2.71% (-3.2 to -2.21) |
| Belgium | 0 (0 to 0) | 0.6 (0.45 to 0.82) | 0 (0 to 0) | 0.35 (0.21 to 0.55) | -0.44% (-0.65 to -0.11) | -2.84% (-3.41 to -2.26) |
| Belize | 0 (0 to 0) | 1.12 (0.87 to 1.42) | 0 (0 to 0) | 3.82 (2.98 to 4.89) | 7.67% (5.28 to 11.11) | 4.57% (3.37 to 5.79) |
| Benin | 2.3 (1.8 to 3) | 112.64 (86.34 to 143.86) | 2.8 (1.7 to 4.2) | 48.48 (30.74 to 72.45) | 0.2% (-0.24 to 0.85) | -2.86% (-3.24 to -2.47) |
| Bermuda | 0 (0 to 0) | 0.84 (0.65 to 1.08) | 0 (0 to 0) | 0.31 (0.23 to 0.42) | -0.72% (-0.81 to -0.6) | -2.97% (-3.3 to -2.63) |
| Bhutan | 0 (0 to 0) | 4.53 (2.64 to 6.39) | 0 (0 to 0) | 1.75 (1.06 to 2.76) | -0.45% (-0.69 to 0.05) | -3.55% (-3.85 to -3.26) |
| Bolivia (Plurinational State of) | 0.1 (0.1 to 0.1) | 3.27 (2.51 to 4.16) | 0.6 (0.4 to 0.9) | 10.4 (6.67 to 14.63) | 5.36% (2.81 to 8.65) | 3.73% (2.73 to 4.74) |
| Bosnia and Herzegovina | 0 (0 to 0) | 0.73 (0.59 to 0.9) | 0 (0 to 0) | 0.15 (0.09 to 0.22) | -0.88% (-0.92 to -0.83) | -7.31% (-8.23 to -6.39) |
| Botswana | 0.5 (0.3 to 0.7) | 69.29 (45.55 to 99.04) | 0.4 (0.2 to 0.6) | 27.4 (15.55 to 42.02) | -0.2% (-0.56 to 0.33) | -2.77% (-3.41 to -2.12) |
| Brazil | 5.6 (4.7 to 6.6) | 7.04 (6 to 8.22) | 2.7 (2.3 to 3.2) | 2.33 (1.98 to 2.77) | -0.52% (-0.62 to -0.38) | -2.67% (-3.41 to -1.92) |
| Brunei Darussalam | 0 (0 to 0) | 10.18 (7.38 to 14.33) | 0 (0 to 0) | 4.72 (3.38 to 6.33) | -0.2% (-0.47 to 0.18) | -2.05% (-2.61 to -1.49) |
| Bulgaria | 0.1 (0.1 to 0.1) | 2.01 (1.66 to 2.44) | 0 (0 to 0) | 0.38 (0.29 to 0.51) | -0.88% (-0.91 to -0.84) | -8.49% (-9.7 to -7.25) |
| Burkina Faso | 2.7 (1.8 to 3.6) | 69.73 (47.58 to 94.76) | 5 (3.3 to 7.3) | 49.34 (32.84 to 71.63) | 0.89% (0.21 to 1.94) | -1.49% (-1.76 to -1.22) |
| Burundi | 1.9 (1.3 to 2.6) | 81.98 (57.11 to 112.91) | 2.5 (1.8 to 3.4) | 48.91 (34.89 to 67.08) | 0.32% (-0.13 to 1.01) | -2.24% (-2.66 to -1.82) |
| Cabo Verde | 0 (0 to 0) | 17.19 (13.03 to 21.76) | 0 (0 to 0) | 5.12 (3.49 to 7.35) | -0.45% (-0.64 to -0.16) | -3.98% (-4.62 to -3.34) |
| Cambodia | 0.4 (0.3 to 0.6) | 7.85 (5.55 to 10.96) | 0.4 (0.3 to 0.6) | 4.52 (3.08 to 6.42) | 0.03% (-0.35 to 0.63) | -1.89% (-2.33 to -1.45) |
| Cameroon | 4.5 (3.4 to 5.8) | 98.18 (74.26 to 125.94) | 9.8 (5.9 to 14.1) | 66.82 (40.65 to 96.68) | 1.16% (0.27 to 2.26) | -0.93% (-1.32 to -0.54) |
| Canada | 0.1 (0.1 to 0.1) | 0.43 (0.33 to 0.53) | 0.1 (0 to 0.1) | 0.31 (0.24 to 0.41) | -0.24% (-0.42 to 0) | -1.1% (-1.61 to -0.58) |
| Central African Republic | 0.9 (0.6 to 1.2) | 70.05 (50.25 to 94.81) | 1.4 (1 to 2.1) | 54.86 (36.37 to 80.11) | 0.62% (0.11 to 1.53) | -0.34% (-0.7 to 0.02) |
| Chad | 2.3 (1.7 to 3) | 86.97 (63.99 to 115.57) | 7.6 (5.2 to 10.2) | 108.11 (74.96 to 144.31) | 2.38% (1.23 to 3.94) | 0.8% (0.54 to 1.06) |
| Chile | 0.2 (0.2 to 0.3) | 3.05 (2.57 to 3.61) | 0.1 (0 to 0.1) | 0.59 (0.43 to 0.84) | -0.75% (-0.82 to -0.65) | -5.36% (-5.9 to -4.82) |
| China | 25.5 (20.4 to 31.4) | 3.71 (2.97 to 4.58) | 4.8 (3.7 to 6) | 0.69 (0.54 to 0.86) | -0.81% (-0.86 to -0.74) | -5.65% (-6.11 to -5.19) |
| Colombia | 0.8 (0.7 to 1) | 4.41 (3.61 to 5.29) | 0.6 (0.4 to 0.8) | 2.36 (1.64 to 3.22) | -0.28% (-0.53 to 0.05) | -2.25% (-2.85 to -1.66) |
| Comoros | 0.1 (0 to 0.1) | 43.18 (10.65 to 68.28) | 0.1 (0 to 0.1) | 19.22 (9.96 to 29.22) | -0.13% (-0.54 to 2.75) | -3.47% (-4.04 to -2.9) |
| Congo | 0.7 (0.5 to 1) | 67.91 (48.64 to 92.54) | 1.2 (0.8 to 1.8) | 45.17 (29.41 to 64.77) | 0.69% (0.01 to 1.77) | -0.67% (-1.34 to -0.01) |
| Cook Islands | 0 (0 to 0) | 0.98 (0.67 to 1.38) | 0 (0 to 0) | 0.41 (0.24 to 0.59) | -0.61% (-0.77 to -0.39) | -2.73% (-2.98 to -2.49) |
| Costa Rica | 0 (0 to 0) | 0.95 (0.78 to 1.15) | 0 (0 to 0) | 0.42 (0.32 to 0.56) | -0.3% (-0.48 to -0.04) | -4.39% (-5.24 to -3.54) |
| Côte d'Ivoire | 4.5 (3.2 to 6.2) | 87.25 (61.19 to 119.06) | 8.6 (5.5 to 12.6) | 68.14 (43.42 to 99.33) | 0.9% (0.14 to 1.98) | -0.49% (-0.82 to -0.15) |
| Croatia | 0 (0 to 0) | 0.88 (0.73 to 1.07) | 0 (0 to 0) | 0.16 (0.1 to 0.24) | -0.87% (-0.92 to -0.81) | -5.24% (-6.07 to -4.41) |
| Cuba | 0 (0 to 0.1) | 0.71 (0.58 to 0.85) | 0.2 (0.1 to 0.2) | 3.8 (2.82 to 5.03) | 3.05% (1.82 to 4.68) | 6.96% (5.43 to 8.51) |
| Cyprus | 0 (0 to 0) | 0.94 (0.63 to 1.36) | 0 (0 to 0) | 0.15 (0.09 to 0.21) | -0.72% (-0.83 to -0.56) | -6.98% (-9.37 to -4.52) |
| Czechia | 0.1 (0.1 to 0.1) | 1.51 (1.24 to 1.8) | 0 (0 to 0) | 0.24 (0.15 to 0.37) | -0.86% (-0.91 to -0.79) | -5.32% (-6.65 to -3.96) |
| Democratic People's Republic of Korea | 1.6 (1 to 2.4) | 13.7 (8.42 to 21.05) | 0.2 (0.1 to 0.3) | 1.63 (1.01 to 2.48) | -0.86% (-0.92 to -0.76) | -7.77% (-8.04 to -7.49) |
| Democratic Republic of the Congo | 11.9 (8.2 to 15.9) | 72.24 (49.96 to 98.26) | 26.1 (18.8 to 33.8) | 65.97 (47.42 to 86.5) | 1.2% (0.5 to 2.34) | 0.6% (0.22 to 0.97) |
| Denmark | 0 (0 to 0) | 0.37 (0.25 to 0.51) | 0 (0 to 0) | 0.32 (0.18 to 0.54) | -0.16% (-0.44 to 0.23) | -1.65% (-2.32 to -0.97) |
| Djibouti | 0.1 (0.1 to 0.2) | 62.6 (40.57 to 89.31) | 0.3 (0.2 to 0.6) | 54.48 (28.37 to 87.26) | 1.76% (0.38 to 3.85) | -0.52% (-0.74 to -0.3) |
| Dominica | 0 (0 to 0) | 3.81 (2.83 to 5.16) | 0 (0 to 0) | 25.54 (17.31 to 36.07) | 5.19% (2.85 to 8.9) | 7.03% (5.26 to 8.82) |
| Dominican Republic | 0.1 (0.1 to 0.1) | 1.84 (1.46 to 2.32) | 0.2 (0.2 to 0.3) | 3.81 (2.57 to 5.51) | 1.95% (0.83 to 3.62) | 3.11% (2.59 to 3.63) |
| Ecuador | 0 (0 to 0.1) | 0.93 (0.74 to 1.18) | 0.4 (0.3 to 0.6) | 4.3 (3.11 to 5.99) | 7.19% (4.62 to 11.13) | 5.85% (4.62 to 7.11) |
| Egypt | 0.6 (0.5 to 0.7) | 2.2 (1.76 to 2.7) | 0.4 (0.3 to 0.6) | 0.82 (0.58 to 1.18) | -0.28% (-0.51 to 0.03) | -2.91% (-3.08 to -2.74) |
| El Salvador | 0.1 (0.1 to 0.2) | 4.89 (3.99 to 5.94) | 0.1 (0.1 to 0.2) | 2.83 (1.87 to 4.31) | -0.26% (-0.54 to 0.15) | -1.37% (-1.99 to -0.74) |
| Equatorial Guinea | 0.1 (0.1 to 0.2) | 71.17 (47.82 to 99.11) | 0.2 (0.1 to 0.3) | 27.08 (14.49 to 46.95) | 0.34% (-0.33 to 1.47) | -3.08% (-3.57 to -2.58) |
| Eritrea | 0.8 (0.5 to 1.1) | 60.16 (38.91 to 85.57) | 2 (1.3 to 3) | 60.07 (39.82 to 90.13) | 1.55% (0.56 to 3.18) | -0.12% (-0.7 to 0.46) |
| Estonia | 0 (0 to 0) | 3.92 (3.1 to 5) | 0 (0 to 0) | 0.37 (0.2 to 0.63) | -0.92% (-0.96 to -0.87) | -8.78% (-10.14 to -7.39) |
| Eswatini | 0.2 (0.1 to 0.2) | 50.79 (35.12 to 68.97) | 0.1 (0 to 0.1) | 13.93 (7.02 to 24.25) | -0.52% (-0.76 to -0.13) | -4.31% (-5.03 to -3.59) |
| Ethiopia | 12.7 (9.3 to 17) | 64.57 (47.34 to 86.71) | 15.5 (11.1 to 22) | 33.41 (23.85 to 46.79) | 0.22% (-0.26 to 0.93) | -2.63% (-2.8 to -2.46) |
| Fiji | 0.1 (0 to 0.1) | 16.9 (12.02 to 23.84) | 0 (0 to 0.1) | 9.2 (6.19 to 13.57) | -0.36% (-0.63 to 0.1) | -2.7% (-3.06 to -2.35) |
| Finland | 0 (0 to 0) | 0.56 (0.44 to 0.72) | 0 (0 to 0) | 0.25 (0.17 to 0.37) | -0.58% (-0.68 to -0.47) | -2.62% (-3.1 to -2.14) |
| France | 0.2 (0.2 to 0.3) | 0.68 (0.54 to 0.85) | 0.1 (0.1 to 0.2) | 0.43 (0.31 to 0.58) | -0.41% (-0.55 to -0.27) | -1.76% (-2.16 to -1.37) |
| Gabon | 0.3 (0.2 to 0.4) | 73.07 (54.45 to 96.1) | 0.3 (0.2 to 0.4) | 29.25 (18.11 to 44.2) | -0.08% (-0.43 to 0.45) | -3.31% (-3.83 to -2.79) |
| Gambia | 0.4 (0.3 to 0.6) | 104.44 (69.77 to 147.03) | 0.8 (0.6 to 1.2) | 78.29 (52.46 to 110.64) | 0.91% (0.23 to 2.18) | -1.07% (-1.5 to -0.64) |
| Georgia | 0 (0 to 0) | 0.6 (0.46 to 0.75) | 0 (0 to 0) | 0.73 (0.55 to 0.95) | -0.32% (-0.5 to -0.07) | 3.52% (2.43 to 4.62) |
| Germany | 0.2 (0.1 to 0.2) | 0.39 (0.33 to 0.47) | 0.1 (0 to 0.1) | 0.16 (0.12 to 0.21) | -0.65% (-0.73 to -0.55) | -3.43% (-3.82 to -3.05) |
| Ghana | 4 (2.8 to 5.3) | 56.62 (38.88 to 74.5) | 5.3 (3.4 to 7.3) | 29.93 (19.48 to 40.58) | 0.33% (-0.21 to 1.07) | -1.48% (-1.79 to -1.17) |
| Greece | 0 (0 to 0) | 0.59 (0.49 to 0.7) | 0 (0 to 0) | 0.36 (0.27 to 0.47) | -0.47% (-0.58 to -0.33) | -1.88% (-2.77 to -0.98) |
| Greenland | 0 (0 to 0) | 1.39 (0.91 to 2.08) | 0 (0 to 0) | 0.42 (0.29 to 0.59) | -0.77% (-0.86 to -0.61) | -6.29% (-7.45 to -5.11) |
| Grenada | 0 (0 to 0) | 3.13 (2.49 to 3.89) | 0 (0 to 0) | 4.29 (3.41 to 5.41) | 0.72% (0.25 to 1.36) | 2.51% (1.13 to 3.91) |
| Guam | 0 (0 to 0) | 0.56 (0.39 to 0.75) | 0 (0 to 0) | 2.24 (1.63 to 3.01) | 3.14% (1.66 to 5.32) | 5.68% (5.26 to 6.1) |
| Guatemala | 0.2 (0.2 to 0.3) | 6.35 (4.87 to 8.05) | 0.3 (0.2 to 0.4) | 2.88 (2.06 to 3.95) | 0.27% (-0.13 to 0.89) | -3.81% (-4.86 to -2.76) |
| Guinea | 3.2 (2.4 to 4.2) | 115.82 (86.09 to 150.5) | 5 (3.5 to 6.9) | 82.42 (58.23 to 111.78) | 0.57% (0.05 to 1.25) | -1.03% (-1.23 to -0.82) |
| Guinea-Bissau | 0.5 (0.3 to 0.7) | 101.37 (70.88 to 138.05) | 0.4 (0.3 to 0.6) | 43.17 (29.12 to 61.46) | -0.1% (-0.43 to 0.38) | -2.82% (-3.11 to -2.52) |
| Guyana | 0 (0 to 0) | 8.24 (6.06 to 10.72) | 0.1 (0.1 to 0.2) | 29.49 (19.84 to 42.31) | 2.56% (1.23 to 4.6) | 4.59% (3.29 to 5.91) |
| Haiti | 0.3 (0.2 to 0.4) | 8.75 (6.12 to 12.29) | 2.1 (1.4 to 3) | 29.77 (19.79 to 41.62) | 6.7% (4.11 to 10.46) | 5.32% (4.32 to 6.33) |
| Honduras | 0.4 (0.3 to 0.5) | 18.07 (13.87 to 23.14) | 0.1 (0.1 to 0.2) | 2.43 (1.45 to 3.69) | -0.67% (-0.8 to -0.47) | -7.27% (-7.61 to -6.92) |
| Hungary | 0.1 (0.1 to 0.2) | 2.65 (2.16 to 3.2) | 0 (0 to 0) | 0.25 (0.18 to 0.34) | -0.92% (-0.95 to -0.89) | -7.14% (-8.48 to -5.78) |
| Iceland | 0 (0 to 0) | 0.34 (0.23 to 0.5) | 0 (0 to 0) | 0.21 (0.13 to 0.34) | -0.26% (-0.41 to -0.13) | -1.3% (-1.52 to -1.08) |
| India | 108.2 (86.5 to 134.5) | 26.23 (21.19 to 32.54) | 76.6 (56.6 to 99.4) | 10.22 (7.58 to 13.21) | -0.29% (-0.5 to -0.04) | -3.6% (-3.94 to -3.26) |
| Indonesia | 10.5 (8.2 to 13.4) | 11.25 (8.79 to 14.4) | 4.4 (3.3 to 5.8) | 3.09 (2.34 to 4.11) | -0.58% (-0.72 to -0.38) | -4.85% (-5.35 to -4.34) |
| Iran (Islamic Republic of) | 1.1 (0.9 to 1.3) | 4.23 (3.4 to 5.14) | 0.3 (0.3 to 0.4) | 0.66 (0.54 to 0.79) | -0.71% (-0.78 to -0.61) | -6.67% (-7.1 to -6.23) |
| Iraq | 0.5 (0.4 to 0.7) | 7.44 (5.15 to 10.3) | 0.5 (0.3 to 0.7) | 2.24 (1.48 to 3.37) | -0.09% (-0.46 to 0.53) | -4.41% (-4.89 to -3.92) |
| Ireland | 0 (0 to 0) | 0.69 (0.53 to 0.89) | 0 (0 to 0) | 0.41 (0.29 to 0.57) | -0.19% (-0.39 to 0.05) | -1.21% (-2.31 to -0.1) |
| Israel | 0 (0 to 0) | 1.18 (0.96 to 1.49) | 0 (0 to 0) | 0.54 (0.33 to 0.84) | -0.2% (-0.49 to 0.18) | -2.97% (-3.69 to -2.24) |
| Italy | 0.2 (0.2 to 0.3) | 0.76 (0.64 to 0.9) | 0.1 (0 to 0.1) | 0.25 (0.18 to 0.34) | -0.73% (-0.8 to -0.64) | -3.54% (-3.92 to -3.17) |
| Jamaica | 0 (0 to 0) | 0.57 (0.46 to 0.7) | 0.1 (0 to 0.1) | 3.51 (2.35 to 5.01) | 6.54% (3.71 to 10.74) | 5.9% (3.57 to 8.27) |
| Japan | 0.5 (0.4 to 0.5) | 0.76 (0.63 to 0.91) | 0.1 (0.1 to 0.1) | 0.18 (0.14 to 0.23) | -0.82% (-0.86 to -0.76) | -5.08% (-5.34 to -4.83) |
| Jordan | 0.1 (0 to 0.1) | 3.11 (2.28 to 4.08) | 0 (0 to 0) | 0.58 (0.42 to 0.78) | -0.34% (-0.57 to 0) | -6.96% (-7.46 to -6.45) |
| Kazakhstan | 0.1 (0.1 to 0.1) | 0.93 (0.75 to 1.15) | 0 (0 to 0.1) | 0.49 (0.36 to 0.65) | -0.42% (-0.55 to -0.26) | -2.62% (-3.21 to -2.02) |
| Kenya | 4.1 (3 to 5.3) | 45.11 (33.74 to 58.57) | 6.8 (4.7 to 9.4) | 27.79 (19.11 to 38.59) | 0.67% (0.15 to 1.51) | -1.43% (-1.96 to -0.9) |
| Kiribati | 0 (0 to 0) | 50.39 (35.5 to 69.99) | 0.1 (0 to 0.1) | 81.09 (55.8 to 111.53) | 1.6% (0.63 to 3.14) | 1.79% (1.05 to 2.55) |
| Kuwait | 0 (0 to 0) | 0.42 (0.35 to 0.52) | 0 (0 to 0) | 0.16 (0.12 to 0.21) | 0.32% (-0.01 to 0.74) | -4.61% (-5.86 to -3.35) |
| Kyrgyzstan | 0 (0 to 0) | 1.61 (1.3 to 1.93) | 0 (0 to 0) | 0.85 (0.68 to 1.07) | -0.19% (-0.36 to 0.02) | -0.92% (-1.47 to -0.37) |
| Lao People's Democratic Republic | 0.1 (0.1 to 0.2) | 5.37 (3.86 to 7.8) | 0.1 (0.1 to 0.1) | 2.16 (1.43 to 2.93) | -0.19% (-0.5 to 0.22) | -3.52% (-3.88 to -3.15) |
| Latvia | 0 (0 to 0) | 1.22 (0.93 to 1.57) | 0 (0 to 0) | 0.57 (0.34 to 0.89) | -0.7% (-0.83 to -0.54) | -2.47% (-3.35 to -1.58) |
| Lebanon | 0.1 (0 to 0.1) | 3.29 (2.24 to 4.53) | 0 (0 to 0) | 0.77 (0.54 to 1.09) | -0.59% (-0.74 to -0.34) | -5% (-5.26 to -4.74) |
| Lesotho | 0.4 (0.3 to 0.6) | 55.4 (36.6 to 76.06) | 0.6 (0.3 to 0.9) | 49.2 (28.07 to 75.91) | 0.25% (-0.33 to 1.17) | 1.43% (0.62 to 2.25) |
| Liberia | 0.9 (0.7 to 1.2) | 110.58 (81.36 to 145.6) | 1.6 (1.1 to 2.2) | 67.34 (45.89 to 92.43) | 0.76% (0.09 to 1.7) | -1.21% (-1.54 to -0.88) |
| Libya | 0 (0 to 0.1) | 2.79 (1.97 to 3.83) | 0 (0 to 0) | 0.61 (0.42 to 0.88) | -0.43% (-0.65 to -0.09) | -4.36% (-5.02 to -3.69) |
| Lithuania | 0 (0 to 0) | 1.67 (1.35 to 2.03) | 0 (0 to 0) | 0.39 (0.23 to 0.63) | -0.85% (-0.91 to -0.76) | -4.57% (-5.65 to -3.48) |
| Luxembourg | 0 (0 to 0) | 0.73 (0.59 to 0.91) | 0 (0 to 0) | 0.39 (0.28 to 0.52) | -0.21% (-0.4 to 0.02) | -2.42% (-2.64 to -2.19) |
| Madagascar | 4.7 (3.6 to 5.8) | 90.79 (71.07 to 113.5) | 7.4 (5.4 to 9.8) | 58.79 (42.44 to 79.46) | 0.59% (0.08 to 1.24) | -1.81% (-2.19 to -1.42) |
| Malawi | 3.4 (2.5 to 4.5) | 85.71 (62.9 to 112.4) | 3.3 (2.1 to 4.7) | 37.64 (23.83 to 54.99) | -0.05% (-0.43 to 0.5) | -2.7% (-3.13 to -2.26) |
| Malaysia | 0.6 (0.4 to 0.7) | 6.24 (4.83 to 8) | 0.5 (0.3 to 0.7) | 2.67 (1.83 to 3.84) | -0.2% (-0.5 to 0.3) | -3.19% (-3.61 to -2.76) |
| Maldives | 0 (0 to 0) | 6.24 (4.6 to 8.62) | 0 (0 to 0) | 0.96 (0.71 to 1.26) | -0.62% (-0.75 to -0.42) | -6.41% (-7.05 to -5.78) |
| Mali | 3.1 (2.5 to 4) | 81.22 (63.28 to 104.47) | 5 (3.3 to 7.2) | 49.65 (33 to 70.47) | 0.6% (0.03 to 1.33) | -2.14% (-2.42 to -1.87) |
| Malta | 0 (0 to 0) | 1.37 (1.08 to 1.67) | 0 (0 to 0) | 0.62 (0.49 to 0.76) | -0.54% (-0.66 to -0.38) | -2.27% (-2.61 to -1.92) |
| Marshall Islands | 0 (0 to 0) | 15.77 (11.15 to 20.79) | 0 (0 to 0) | 11.34 (6.68 to 17.13) | 0.08% (-0.41 to 0.75) | -1.35% (-1.63 to -1.06) |
| Mauritania | 1.6 (1.2 to 2) | 183.16 (143.04 to 234.67) | 1.9 (1.2 to 2.9) | 103.08 (65.1 to 152.99) | 0.23% (-0.24 to 0.91) | -1.42% (-1.65 to -1.19) |
| Mauritius | 0 (0 to 0) | 1.7 (1.37 to 2.08) | 0 (0 to 0) | 1.92 (1.37 to 2.61) | 0.12% (-0.25 to 0.61) | 1.03% (-0.31 to 2.4) |
| Mexico | 2.6 (2.2 to 3) | 5.66 (4.82 to 6.61) | 1.4 (1.1 to 1.8) | 2.08 (1.59 to 2.69) | -0.45% (-0.6 to -0.26) | -3.68% (-4.12 to -3.24) |
| Micronesia (Federated States of) | 0 (0 to 0) | 20.37 (13.56 to 29.59) | 0 (0 to 0) | 10.18 (3.83 to 16.56) | -0.44% (-0.78 to -0.04) | -2.79% (-2.94 to -2.65) |
| Monaco | 0 (0 to 0) | 0.28 (0.18 to 0.4) | 0 (0 to 0) | 0.21 (0.12 to 0.33) | -0.25% (-0.43 to -0.07) | -1.02% (-1.18 to -0.87) |
| Mongolia | 0 (0 to 0) | 3.37 (2.43 to 4.39) | 0 (0 to 0) | 1.48 (0.99 to 2.08) | -0.26% (-0.53 to 0.13) | -2.94% (-3.45 to -2.43) |
| Montenegro | 0 (0 to 0) | 0.26 (0.17 to 0.37) | 0 (0 to 0) | 0.2 (0.13 to 0.3) | -0.31% (-0.44 to -0.15) | -0.85% (-1.07 to -0.63) |
| Morocco | 0.6 (0.4 to 0.8) | 4.61 (3.47 to 6.03) | 0.3 (0.2 to 0.5) | 1.72 (1.18 to 2.55) | -0.44% (-0.64 to -0.12) | -3.69% (-4.11 to -3.27) |
| Mozambique | 2.6 (1.9 to 3.6) | 45.77 (32.86 to 62.34) | 6.1 (3.8 to 8.8) | 45.62 (27.92 to 66.01) | 1.31% (0.36 to 2.54) | 1.25% (0.72 to 1.79) |
| Myanmar | 1.2 (0.8 to 1.8) | 5.51 (3.77 to 8.07) | 2.6 (1.7 to 3.8) | 8.7 (5.65 to 12.59) | 1.14% (0.29 to 2.58) | 1.49% (1.17 to 1.82) |
| Namibia | 0.3 (0.2 to 0.4) | 46.82 (28.01 to 63.72) | 0.3 (0.2 to 0.5) | 23.43 (13.09 to 38.44) | 0.03% (-0.44 to 1.12) | -2.01% (-2.37 to -1.66) |
| Nauru | 0 (0 to 0) | 24.94 (16.67 to 36.87) | 0 (0 to 0) | 13.39 (8.29 to 21.43) | -0.39% (-0.64 to 0.05) | -2.37% (-2.73 to -2.01) |
| Nepal | 0.4 (0.3 to 0.6) | 4.51 (3.37 to 5.94) | 0.2 (0.1 to 0.2) | 0.84 (0.57 to 1.15) | -0.64% (-0.77 to -0.47) | -6.09% (-6.6 to -5.57) |
| Netherlands | 0.1 (0 to 0.1) | 0.69 (0.55 to 0.84) | 0 (0 to 0) | 0.28 (0.19 to 0.41) | -0.64% (-0.74 to -0.53) | -3.83% (-4.24 to -3.42) |
| New Zealand | 0 (0 to 0) | 0.53 (0.38 to 0.72) | 0 (0 to 0) | 0.45 (0.3 to 0.65) | -0.11% (-0.35 to 0.2) | -0.97% (-1.34 to -0.59) |
| Nicaragua | 0 (0 to 0) | 0.9 (0.71 to 1.14) | 0 (0 to 0) | 0.44 (0.32 to 0.58) | -0.05% (-0.29 to 0.25) | -1.88% (-2.61 to -1.16) |
| Niger | 2.5 (1.8 to 3.3) | 75.4 (53.96 to 99.19) | 6.9 (4.4 to 9.7) | 74.83 (47.56 to 105.25) | 1.75% (0.72 to 3.14) | -0.31% (-0.55 to -0.07) |
| Nigeria | 24.7 (15.4 to 38.3) | 62.21 (39.15 to 97.01) | 42 (25.6 to 68.9) | 39.48 (23.94 to 64.84) | 0.7% (-0.03 to 1.98) | -1.76% (-2.3 to -1.22) |
| Niue | 0 (0 to 0) | 10.58 (6.74 to 15.91) | 0 (0 to 0) | 4.91 (2.58 to 7.86) | -0.65% (-0.83 to -0.37) | -3.23% (-3.42 to -3.04) |
| North Macedonia | 0 (0 to 0) | 0.99 (0.76 to 1.25) | 0 (0 to 0) | 0.29 (0.21 to 0.4) | -0.71% (-0.8 to -0.58) | -4.97% (-5.39 to -4.55) |
| Northern Mariana Islands | 0 (0 to 0) | 8.47 (5.6 to 12.13) | 0 (0 to 0) | 8.1 (5.46 to 11.62) | -0.35% (-0.62 to 0.12) | -0.16% (-0.83 to 0.51) |
| Norway | 0 (0 to 0) | 0.53 (0.42 to 0.66) | 0 (0 to 0) | 0.18 (0.11 to 0.3) | -0.6% (-0.73 to -0.45) | -3.5% (-4.37 to -2.63) |
| Oman | 0 (0 to 0) | 3.42 (2.39 to 4.8) | 0 (0 to 0) | 0.61 (0.45 to 0.81) | -0.44% (-0.65 to -0.15) | -6.07% (-6.37 to -5.76) |
| Pakistan | 11.9 (9.1 to 15.2) | 24.7 (19.17 to 31.25) | 27.6 (19.4 to 37.4) | 23.65 (16.49 to 32.05) | 1.32% (0.54 to 2.37) | -0.32% (-0.74 to 0.11) |
| Palau | 0 (0 to 0) | 2.29 (1.5 to 3.37) | 0 (0 to 0) | 1.3 (0.83 to 1.96) | -0.56% (-0.74 to -0.24) | -1.86% (-1.93 to -1.79) |
| Palestine | 0 (0 to 0) | 3.53 (2.48 to 5.04) | 0 (0 to 0) | 0.67 (0.52 to 0.86) | -0.44% (-0.64 to -0.15) | -5.24% (-5.6 to -4.89) |
| Panama | 0 (0 to 0) | 3.12 (2.59 to 3.77) | 0.1 (0.1 to 0.1) | 4.42 (3.07 to 6.15) | 1.28% (0.52 to 2.27) | 1.8% (1.33 to 2.27) |
| Papua New Guinea | 0.2 (0.2 to 0.3) | 11.52 (8.01 to 15.34) | 0.5 (0.4 to 0.7) | 10.19 (7.37 to 14.32) | 1.32% (0.54 to 2.53) | -0.52% (-0.81 to -0.22) |
| Paraguay | 0 (0 to 0) | 0.79 (0.63 to 0.97) | 0.1 (0.1 to 0.1) | 2.04 (1.39 to 2.97) | 4.05% (2.29 to 6.73) | 3.08% (1.52 to 4.67) |
| Peru | 0.4 (0.3 to 0.5) | 3.43 (2.75 to 4.33) | 0.7 (0.5 to 1) | 3.81 (2.57 to 5.58) | 0.76% (0.16 to 1.74) | 0.06% (-1.04 to 1.17) |
| Philippines | 3.7 (3 to 4.5) | 11.42 (9.13 to 14.02) | 4 (2.8 to 5.6) | 6.77 (4.75 to 9.55) | 0.08% (-0.27 to 0.56) | -1.47% (-1.61 to -1.34) |
| Poland | 0.3 (0.3 to 0.4) | 1.6 (1.34 to 1.88) | 0 (0 to 0) | 0.12 (0.09 to 0.16) | -0.93% (-0.95 to -0.9) | -9.4% (-10.64 to -8.14) |
| Portugal | 0.1 (0 to 0.1) | 1.1 (0.9 to 1.33) | 0 (0 to 0) | 0.2 (0.15 to 0.26) | -0.84% (-0.88 to -0.79) | -6.88% (-7.82 to -5.92) |
| Puerto Rico | 0 (0 to 0) | 1.53 (1.28 to 1.85) | 0 (0 to 0) | 0.46 (0.33 to 0.62) | -0.74% (-0.82 to -0.63) | -4.69% (-5.64 to -3.74) |
| Qatar | 0 (0 to 0) | 3.07 (2.16 to 4.15) | 0 (0 to 0) | 0.48 (0.34 to 0.67) | -0.09% (-0.43 to 0.44) | -6.32% (-6.69 to -5.95) |
| Republic of Korea | 0.1 (0.1 to 0.2) | 0.49 (0.4 to 0.61) | 0 (0 to 0.1) | 0.17 (0.13 to 0.22) | -0.71% (-0.78 to -0.59) | -4.47% (-5.01 to -3.93) |
| Republic of Moldova | 0 (0 to 0) | 1.1 (0.82 to 1.44) | 0 (0 to 0) | 0.44 (0.32 to 0.6) | -0.69% (-0.77 to -0.6) | -2.94% (-3.42 to -2.46) |
| Romania | 0 (0 to 0.1) | 0.44 (0.33 to 0.58) | 0 (0 to 0) | 0.5 (0.37 to 0.66) | -0.22% (-0.4 to 0.02) | 0.42% (-0.24 to 1.08) |
| Russian Federation | 6.4 (5.5 to 7.5) | 8.18 (6.95 to 9.61) | 0.7 (0.5 to 0.9) | 0.95 (0.71 to 1.25) | -0.9% (-0.93 to -0.86) | -8.31% (-8.69 to -7.93) |
| Rwanda | 1 (0.8 to 1.4) | 35.05 (26.39 to 47.4) | 0.9 (0.6 to 1.2) | 13.88 (9.01 to 20.14) | -0.15% (-0.52 to 0.38) | -3.28% (-3.69 to -2.87) |
| Saint Kitts and Nevis | 0 (0 to 0) | 1.09 (0.86 to 1.38) | 0 (0 to 0) | 1.44 (0.55 to 2.36) | 0.82% (-0.36 to 2.17) | 1.56% (0.9 to 2.21) |
| Saint Lucia | 0 (0 to 0) | 0.88 (0.72 to 1.08) | 0 (0 to 0) | 0.95 (0.72 to 1.22) | 0.33% (-0.04 to 0.81) | 1.19% (0.71 to 1.68) |
| Saint Vincent and the Grenadines | 0 (0 to 0) | 10.61 (8.71 to 12.75) | 0 (0 to 0) | 14.43 (11.07 to 18.5) | 0.34% (-0.05 to 0.88) | 1.25% (0.94 to 1.57) |
| Samoa | 0 (0 to 0) | 6.17 (4.01 to 9.13) | 0 (0 to 0) | 2.43 (1.15 to 3.86) | -0.47% (-0.78 to -0.01) | -4.1% (-4.59 to -3.62) |
| San Marino | 0 (0 to 0) | 0.35 (0.24 to 0.48) | 0 (0 to 0) | 0.29 (0.19 to 0.43) | 0.05% (-0.28 to 0.48) | -0.6% (-0.67 to -0.53) |
| Sao Tome and Principe | 0 (0 to 0) | 42.59 (21.81 to 62.57) | 0 (0 to 0) | 17.24 (11.7 to 24.49) | -0.1% (-0.48 to 0.99) | -4.05% (-4.8 to -3.3) |
| Saudi Arabia | 0.2 (0.1 to 0.3) | 3.09 (2.09 to 4.36) | 0.3 (0.2 to 0.4) | 1.23 (0.84 to 1.76) | 0.37% (-0.16 to 1.23) | -2.35% (-2.75 to -1.96) |
| Senegal | 3.1 (2.3 to 4.1) | 94.79 (70.03 to 124.46) | 6.2 (4.1 to 8.5) | 85.23 (56.74 to 117.63) | 0.98% (0.25 to 2.02) | 0.24% (-0.03 to 0.5) |
| Serbia | 0 (0 to 0) | 0.47 (0.35 to 0.63) | 0 (0 to 0) | 0.21 (0.15 to 0.3) | -0.61% (-0.73 to -0.5) | -2.78% (-3.14 to -2.41) |
| Seychelles | 0 (0 to 0) | 7.12 (5.49 to 9.03) | 0 (0 to 0) | 3.42 (2.54 to 4.55) | -0.4% (-0.59 to -0.16) | -1.82% (-2.27 to -1.36) |
| Sierra Leone | 1.2 (0.8 to 1.7) | 71.2 (48.64 to 97.94) | 3.6 (2.4 to 4.9) | 84.21 (57.38 to 115.34) | 1.95% (0.84 to 3.54) | 1.17% (0.82 to 1.53) |
| Singapore | 0 (0 to 0) | 1.22 (0.99 to 1.49) | 0 (0 to 0) | 0.14 (0.11 to 0.18) | -0.81% (-0.87 to -0.74) | -6.77% (-7.79 to -5.73) |
| Slovakia | 0 (0 to 0) | 0.78 (0.59 to 1.01) | 0 (0 to 0) | 0.3 (0.21 to 0.43) | -0.63% (-0.74 to -0.5) | -2.57% (-3.54 to -1.59) |
| Slovenia | 0 (0 to 0) | 1.41 (0.98 to 1.99) | 0 (0 to 0) | 0.24 (0.16 to 0.33) | -0.87% (-0.92 to -0.79) | -4.22% (-5.65 to -2.77) |
| Solomon Islands | 0.1 (0 to 0.1) | 36.18 (23.36 to 54.55) | 0.1 (0.1 to 0.2) | 35.4 (22.04 to 51.37) | 1.2% (0.29 to 2.68) | -0.11% (-0.36 to 0.13) |
| Somalia | 1.7 (1 to 2.5) | 60.53 (36.33 to 91.65) | 4.7 (3 to 6.7) | 57.13 (36.63 to 83.6) | 1.72% (0.8 to 3.29) | 0.07% (-0.1 to 0.24) |
| South Africa | 10.8 (8.7 to 13.2) | 54.37 (43.67 to 66.02) | 4.1 (2.5 to 6.3) | 12.9 (7.89 to 19.3) | -0.62% (-0.77 to -0.4) | -4.02% (-5.47 to -2.54) |
| South Sudan | 0.8 (0.5 to 1.2) | 34.06 (21.44 to 50.98) | 1.1 (0.6 to 1.8) | 27.09 (15.24 to 45.53) | 0.44% (-0.15 to 1.46) | -0.43% (-0.7 to -0.16) |
| Spain | 0.1 (0.1 to 0.1) | 0.42 (0.33 to 0.53) | 0 (0 to 0.1) | 0.22 (0.14 to 0.32) | -0.45% (-0.6 to -0.31) | -1.54% (-2 to -1.08) |
| Sri Lanka | 1.7 (1.3 to 2.2) | 17.89 (13.62 to 22.93) | 1.3 (0.8 to 1.8) | 11.66 (7.8 to 16.85) | -0.27% (-0.54 to 0.16) | -1.43% (-1.61 to -1.24) |
| Sudan | 0.4 (0.3 to 0.6) | 4.53 (3.28 to 6.03) | 0.4 (0.2 to 0.6) | 1.89 (1.12 to 2.85) | -0.05% (-0.45 to 0.55) | -2.57% (-2.93 to -2.2) |
| Suriname | 0 (0 to 0) | 12.93 (8 to 17.15) | 0.1 (0 to 0.1) | 18.79 (13.18 to 25.41) | 1.07% (0.3 to 2.49) | 1.58% (1.14 to 2.03) |
| Sweden | 0 (0 to 0) | 0.6 (0.39 to 0.9) | 0 (0 to 0) | 0.38 (0.22 to 0.61) | -0.29% (-0.52 to 0.06) | -1.57% (-1.95 to -1.19) |
| Switzerland | 0 (0 to 0) | 0.42 (0.31 to 0.56) | 0 (0 to 0) | 0.3 (0.21 to 0.44) | -0.2% (-0.38 to 0) | -1.9% (-2.49 to -1.31) |
| Syrian Arab Republic | 0.1 (0.1 to 0.2) | 2.59 (1.87 to 3.47) | 0 (0 to 0) | 0.39 (0.27 to 0.55) | -0.79% (-0.87 to -0.69) | -6.6% (-7.05 to -6.15) |
| Taiwan (Province of China) | 0.2 (0.2 to 0.2) | 1.54 (1.27 to 1.87) | 0 (0 to 0.1) | 0.41 (0.29 to 0.54) | -0.73% (-0.81 to -0.63) | -4.71% (-5.37 to -4.03) |
| Tajikistan | 0.1 (0.1 to 0.1) | 3.55 (2.75 to 4.44) | 0 (0 to 0.1) | 0.83 (0.57 to 1.18) | -0.53% (-0.68 to -0.31) | -6% (-6.7 to -5.3) |
| Thailand | 0.5 (0.3 to 0.7) | 1.39 (0.98 to 1.94) | 0.4 (0.3 to 0.6) | 1.37 (0.87 to 1.96) | -0.08% (-0.45 to 0.5) | -0.47% (-1.35 to 0.43) |
| Timor-Leste | 0.1 (0 to 0.1) | 15.72 (9.5 to 22.22) | 0.1 (0 to 0.1) | 13.88 (3.1 to 20.46) | 0.44% (-0.64 to 1.67) | -1.16% (-1.8 to -0.51) |
| Togo | 1.3 (1 to 1.8) | 83.81 (62.02 to 110.24) | 1.4 (0.9 to 2) | 35.12 (22.48 to 49.28) | 0.06% (-0.34 to 0.57) | -3.15% (-3.62 to -2.69) |
| Tokelau | 0 (0 to 0) | 16.19 (10.2 to 24.6) | 0 (0 to 0) | 8.04 (4.73 to 12.5) | -0.56% (-0.75 to -0.19) | -2.62% (-2.71 to -2.53) |
| Tonga | 0 (0 to 0) | 13.1 (9.72 to 17.28) | 0 (0 to 0) | 8.48 (5.35 to 12.52) | -0.22% (-0.54 to 0.25) | -1.9% (-2.07 to -1.73) |
| Trinidad and Tobago | 0 (0 to 0) | 1.33 (1.08 to 1.63) | 0 (0 to 0.1) | 4.75 (3.05 to 7.1) | 2.92% (1.41 to 5.26) | 5.55% (4.22 to 6.89) |
| Tunisia | 0.1 (0.1 to 0.1) | 1.99 (1.51 to 2.59) | 0 (0 to 0.1) | 0.65 (0.42 to 0.93) | -0.54% (-0.71 to -0.27) | -3.91% (-4.06 to -3.75) |
| Turkey | 0.8 (0.6 to 1) | 2.47 (1.88 to 3.2) | 0.2 (0.1 to 0.2) | 0.4 (0.3 to 0.52) | -0.77% (-0.84 to -0.66) | -7.52% (-8.39 to -6.65) |
| Turkmenistan | 0 (0 to 0.1) | 2.74 (2.2 to 3.41) | 0.1 (0 to 0.1) | 2.35 (1.67 to 3.28) | 0.19% (-0.16 to 0.7) | -0.16% (-0.64 to 0.32) |
| Tuvalu | 0 (0 to 0) | 19.78 (13.52 to 28.17) | 0 (0 to 0) | 8.14 (4.97 to 12.21) | -0.54% (-0.74 to -0.21) | -3.31% (-3.44 to -3.19) |
| Uganda | 1.7 (1.1 to 2.5) | 24.77 (15.69 to 36.19) | 5.7 (3.7 to 8.3) | 30.6 (20.3 to 44.46) | 2.31% (0.94 to 4.74) | 0.8% (0.15 to 1.45) |
| Ukraine | 0.2 (0.2 to 0.3) | 0.9 (0.67 to 1.19) | 0.1 (0.1 to 0.1) | 0.47 (0.31 to 0.66) | -0.58% (-0.7 to -0.42) | -1.91% (-2.58 to -1.23) |
| United Arab Emirates | 0 (0 to 0) | 2.25 (1.55 to 3.14) | 0 (0 to 0) | 0.44 (0.28 to 0.63) | 0.02% (-0.4 to 0.69) | -6.03% (-6.47 to -5.58) |
| United Kingdom | 0.4 (0.3 to 0.5) | 1.33 (1.1 to 1.58) | 0.1 (0.1 to 0.2) | 0.42 (0.31 to 0.55) | -0.66% (-0.74 to -0.56) | -4.23% (-4.54 to -3.91) |
| United Republic of Tanzania | 9.1 (6.6 to 12.1) | 87.76 (64.13 to 118.09) | 13.7 (9.3 to 19) | 52.86 (35.4 to 73.7) | 0.51% (-0.01 to 1.38) | -0.99% (-1.37 to -0.61) |
| United States of America | 2.6 (2.2 to 3.1) | 1.93 (1.61 to 2.27) | 1.2 (1 to 1.5) | 0.83 (0.68 to 1) | -0.53% (-0.63 to -0.4) | -2.45% (-2.76 to -2.14) |
| United States Virgin Islands | 0 (0 to 0) | 1.08 (0.8 to 1.4) | 0 (0 to 0) | 0.76 (0.51 to 1.17) | -0.45% (-0.65 to -0.11) | -0.68% (-1.3 to -0.05) |
| Uruguay | 0 (0 to 0) | 1.22 (0.96 to 1.57) | 0 (0 to 0) | 0.58 (0.42 to 0.81) | -0.48% (-0.62 to -0.29) | -3.7% (-4.27 to -3.13) |
| Uzbekistan | 0.1 (0.1 to 0.1) | 1.04 (0.79 to 1.37) | 0.1 (0.1 to 0.2) | 0.66 (0.5 to 0.85) | 0.13% (-0.15 to 0.52) | -0.88% (-1.38 to -0.37) |
| Vanuatu | 0 (0 to 0) | 8.42 (4.91 to 13.17) | 0 (0 to 0) | 7.26 (3.72 to 10.97) | 0.82% (-0.14 to 2.33) | -1.27% (-1.58 to -0.96) |
| Venezuela (Bolivarian Republic of) | 0.1 (0.1 to 0.1) | 0.88 (0.72 to 1.06) | 0.6 (0.4 to 0.9) | 4.29 (3 to 5.99) | 5.76% (3.58 to 9.13) | 5.8% (4.97 to 6.64) |
| Viet Nam | 0.3 (0.2 to 0.5) | 0.83 (0.55 to 1.24) | 0.1 (0.1 to 0.2) | 0.27 (0.2 to 0.38) | -0.53% (-0.71 to -0.28) | -3.12% (-3.45 to -2.79) |
| Yemen | 0.3 (0.2 to 0.5) | 6 (3.31 to 9.2) | 0.9 (0.5 to 1.4) | 5.8 (3.24 to 8.8) | 1.78% (0.4 to 4.57) | -0.12% (-0.57 to 0.34) |
| Zambia | 1.8 (1.3 to 2.3) | 58.16 (42.8 to 76.92) | 2.1 (1.4 to 2.9) | 24.95 (16.4 to 35.6) | 0.14% (-0.29 to 0.72) | -3.21% (-3.47 to -2.95) |
| Zimbabwe | 3.1 (2.3 to 3.9) | 67.39 (50.61 to 86.84) | 4.3 (2.9 to 6) | 54.22 (36.68 to 75.34) | 0.41% (-0.08 to 1.13) | 1.57% (0.41 to 2.74) |
