# Supplementary material for: Global burden and trends of ectopic pregnancy: An observational trend study from 1990 to 2019
Source: PLoS One. 2023 Oct 26;18(10):e0291316. doi: 10.1371/journal.pone.0291316 (PMC10602312; doi:10.1371/journal.pone.0291316)
Supplement: S3 Table — (DOCX) [file pone.0291316.s003.docx]

S6 Table. Deaths of ectopic pregnancy in 1990 and 2019 for all locations, with EAPC from 1990 and 2019.

| location | Num_1990 | ASR_1990 | Num_2019 | ASR_2019 | Num_change | EAPC_CI |
| --- | --- | --- | --- | --- | --- | --- |
| Afghanistan | 0.005 (0.004 to 0.007) | 0.115 (0.081 to 0.161) | 0.012 (0.008 to 0.017) | 0.075 (0.051 to 0.11) | 1.24% (0.5 to 2.38) | -1.27% (-1.71 to -0.83) |
| Albania | 0 (0 to 0) | 0.021 (0.016 to 0.027) | 0 (0 to 0) | 0.008 (0.005 to 0.011) | -0.74% (-0.85 to -0.59) | -1.94% (-2.43 to -1.46) |
| Algeria | 0.007 (0.005 to 0.009) | 0.059 (0.042 to 0.083) | 0.003 (0.002 to 0.004) | 0.014 (0.01 to 0.019) | -0.51% (-0.68 to -0.22) | -4.99% (-5.2 to -4.77) |
| American Samoa | 0 (0 to 0) | 0.07 (0.05 to 0.095) | 0 (0 to 0) | 0.109 (0.075 to 0.156) | 0.82% (0.07 to 1.96) | 1.37% (0.81 to 1.93) |
| Andorra | 0 (0 to 0) | 0.003 (0.002 to 0.004) | 0 (0 to 0) | 0.001 (0.001 to 0.002) | -0.55% (-0.75 to -0.23) | -3.56% (-3.68 to -3.44) |
| Angola | 0.047 (0.031 to 0.065) | 1.081 (0.707 to 1.509) | 0.131 (0.082 to 0.182) | 0.971 (0.602 to 1.368) | 1.8% (0.64 to 3.45) | 0.05% (-0.22 to 0.33) |
| Antigua and Barbuda | 0 (0 to 0) | 0.029 (0.023 to 0.036) | 0 (0 to 0) | 0.092 (0.071 to 0.118) | 3.41% (2.14 to 5.11) | 4.94% (4.08 to 5.8) |
| Argentina | 0.001 (0.001 to 0.001) | 0.007 (0.006 to 0.009) | 0.006 (0.005 to 0.007) | 0.026 (0.021 to 0.031) | 4.25% (2.95 to 5.86) | 4.62% (3.32 to 5.93) |
| Armenia | 0 (0 to 0) | 0.01 (0.008 to 0.013) | 0 (0 to 0) | 0.003 (0.002 to 0.004) | -0.78% (-0.85 to -0.68) | -3.09% (-4.29 to -1.88) |
| Australia | 0.001 (0.001 to 0.001) | 0.013 (0.01 to 0.016) | 0 (0 to 0.001) | 0.004 (0.003 to 0.005) | -0.61% (-0.72 to -0.45) | -4.15% (-5.03 to -3.26) |
| Austria | 0.001 (0.001 to 0.001) | 0.016 (0.013 to 0.019) | 0 (0 to 0) | 0.001 (0.001 to 0.001) | -0.94% (-0.96 to -0.92) | -11.03% (-12.31 to -9.73) |
| Azerbaijan | 0 (0 to 0) | 0.008 (0.006 to 0.01) | 0 (0 to 0) | 0.003 (0.002 to 0.004) | -0.57% (-0.72 to -0.34) | -4.05% (-4.59 to -3.5) |
| Bahamas | 0 (0 to 0) | 0.078 (0.062 to 0.097) | 0 (0 to 0.001) | 0.195 (0.137 to 0.267) | 2.4% (1.29 to 3.98) | 3.11% (2.17 to 4.05) |
| Bahrain | 0 (0 to 0) | 0.023 (0.017 to 0.03) | 0 (0 to 0) | 0.008 (0.005 to 0.01) | -0.01% (-0.37 to 0.56) | -2.93% (-3.52 to -2.34) |
| Bangladesh | 0.01 (0.007 to 0.012) | 0.018 (0.012 to 0.024) | 0.031 (0.022 to 0.044) | 0.035 (0.024 to 0.049) | 2.28% (1.1 to 4.12) | 5.68% (4.21 to 7.18) |
| Barbados | 0 (0 to 0) | 0.048 (0.039 to 0.059) | 0 (0 to 0) | 0.143 (0.108 to 0.189) | 1.85% (1.02 to 3.04) | 4.59% (3.52 to 5.68) |
| Belarus | 0.001 (0.001 to 0.001) | 0.021 (0.017 to 0.025) | 0 (0 to 0) | 0.006 (0.004 to 0.008) | -0.79% (-0.86 to -0.67) | -4.37% (-4.82 to -3.93) |
| Belgium | 0 (0 to 0) | 0.007 (0.006 to 0.009) | 0 (0 to 0) | 0.002 (0.002 to 0.002) | -0.72% (-0.8 to -0.62) | -5.9% (-6.89 to -4.89) |
| Belize | 0 (0 to 0) | 0.014 (0.011 to 0.018) | 0 (0 to 0) | 0.063 (0.048 to 0.081) | 10.06% (6.74 to 14.74) | 5.2% (3.83 to 6.58) |
| Benin | 0.041 (0.032 to 0.053) | 2.086 (1.607 to 2.684) | 0.049 (0.031 to 0.074) | 0.883 (0.567 to 1.33) | 0.19% (-0.25 to 0.82) | -2.92% (-3.3 to -2.53) |
| Bermuda | 0 (0 to 0) | 0.012 (0.009 to 0.016) | 0 (0 to 0) | 0.004 (0.003 to 0.005) | -0.77% (-0.86 to -0.64) | -3.55% (-3.96 to -3.13) |
| Bhutan | 0 (0 to 0) | 0.071 (0.041 to 0.101) | 0 (0 to 0) | 0.026 (0.015 to 0.042) | -0.45% (-0.71 to 0.07) | -3.68% (-3.99 to -3.36) |
| Bolivia (Plurinational State of) | 0.001 (0.001 to 0.002) | 0.048 (0.036 to 0.062) | 0.011 (0.007 to 0.015) | 0.177 (0.11 to 0.253) | 6.43% (3.35 to 10.69) | 4.14% (3.06 to 5.24) |
| Bosnia and Herzegovina | 0 (0 to 0) | 0.01 (0.008 to 0.013) | 0 (0 to 0) | 0.001 (0 to 0.001) | -0.96% (-0.97 to -0.94) | -11.66% (-12.72 to -10.59) |
| Botswana | 0.008 (0.005 to 0.011) | 1.168 (0.752 to 1.672) | 0.006 (0.003 to 0.01) | 0.444 (0.25 to 0.68) | -0.2% (-0.58 to 0.34) | -2.91% (-3.57 to -2.25) |
| Brazil | 0.095 (0.08 to 0.111) | 0.122 (0.103 to 0.142) | 0.045 (0.038 to 0.053) | 0.038 (0.032 to 0.045) | -0.53% (-0.63 to -0.4) | -2.84% (-3.62 to -2.05) |
| Brunei Darussalam | 0 (0 to 0) | 0.175 (0.126 to 0.248) | 0 (0 to 0) | 0.081 (0.057 to 0.109) | -0.19% (-0.47 to 0.21) | -2.03% (-2.6 to -1.45) |
| Bulgaria | 0.001 (0.001 to 0.002) | 0.034 (0.028 to 0.042) | 0 (0 to 0) | 0.004 (0.003 to 0.006) | -0.92% (-0.95 to -0.89) | -10.56% (-12.01 to -9.09) |
| Burkina Faso | 0.049 (0.033 to 0.067) | 1.333 (0.903 to 1.835) | 0.091 (0.06 to 0.131) | 0.916 (0.604 to 1.34) | 0.84% (0.19 to 1.89) | -1.63% (-1.94 to -1.32) |
| Burundi | 0.034 (0.024 to 0.047) | 1.555 (1.071 to 2.152) | 0.044 (0.032 to 0.06) | 0.915 (0.645 to 1.271) | 0.3% (-0.15 to 1.02) | -2.32% (-2.74 to -1.91) |
| Cabo Verde | 0 (0 to 0.001) | 0.295 (0.222 to 0.374) | 0 (0 to 0) | 0.086 (0.059 to 0.123) | -0.44% (-0.63 to -0.16) | -4.06% (-4.7 to -3.42) |
| Cambodia | 0.007 (0.005 to 0.009) | 0.132 (0.092 to 0.185) | 0.007 (0.005 to 0.01) | 0.078 (0.053 to 0.112) | 0.07% (-0.33 to 0.73) | -1.76% (-2.22 to -1.31) |
| Cameroon | 0.079 (0.059 to 0.101) | 1.783 (1.343 to 2.289) | 0.167 (0.101 to 0.242) | 1.19 (0.73 to 1.744) | 1.13% (0.25 to 2.22) | -1.01% (-1.4 to -0.61) |
| Canada | 0.001 (0.001 to 0.001) | 0.006 (0.004 to 0.007) | 0.001 (0 to 0.001) | 0.004 (0.003 to 0.005) | -0.33% (-0.53 to -0.02) | -1.62% (-2.32 to -0.92) |
| Central African Republic | 0.015 (0.011 to 0.02) | 1.254 (0.898 to 1.706) | 0.025 (0.016 to 0.036) | 0.976 (0.638 to 1.435) | 0.62% (0.09 to 1.51) | -0.37% (-0.73 to -0.01) |
| Chad | 0.039 (0.029 to 0.052) | 1.561 (1.138 to 2.07) | 0.129 (0.089 to 0.174) | 1.923 (1.33 to 2.605) | 2.31% (1.21 to 3.85) | 0.75% (0.49 to 1.01) |
| Chile | 0.003 (0.003 to 0.004) | 0.048 (0.04 to 0.056) | 0.001 (0 to 0.001) | 0.005 (0.004 to 0.007) | -0.85% (-0.89 to -0.8) | -6.72% (-7.54 to -5.91) |
| China | 0.387 (0.304 to 0.481) | 0.057 (0.045 to 0.071) | 0.061 (0.046 to 0.078) | 0.009 (0.007 to 0.011) | -0.84% (-0.89 to -0.78) | -6.38% (-6.92 to -5.83) |
| Colombia | 0.013 (0.011 to 0.016) | 0.07 (0.057 to 0.083) | 0.009 (0.006 to 0.013) | 0.037 (0.025 to 0.051) | -0.28% (-0.54 to 0.07) | -2.32% (-2.94 to -1.69) |
| Comoros | 0.001 (0 to 0.002) | 0.822 (0.209 to 1.297) | 0.001 (0.001 to 0.002) | 0.36 (0.188 to 0.55) | -0.13% (-0.54 to 2.72) | -3.51% (-4.06 to -2.96) |
| Congo | 0.013 (0.009 to 0.017) | 1.218 (0.871 to 1.692) | 0.022 (0.014 to 0.031) | 0.804 (0.514 to 1.167) | 0.72% (0.01 to 1.82) | -0.7% (-1.35 to -0.04) |
| Cook Islands | 0 (0 to 0) | 0.012 (0.008 to 0.019) | 0 (0 to 0) | 0.004 (0.002 to 0.006) | -0.69% (-0.88 to -0.4) | -3.54% (-3.92 to -3.17) |
| Costa Rica | 0 (0 to 0) | 0.013 (0.01 to 0.015) | 0 (0 to 0) | 0.005 (0.004 to 0.007) | -0.33% (-0.54 to 0) | -4.88% (-5.9 to -3.84) |
| Côte d'Ivoire | 0.079 (0.055 to 0.108) | 1.597 (1.124 to 2.188) | 0.15 (0.096 to 0.219) | 1.229 (0.77 to 1.8) | 0.91% (0.15 to 2.03) | -0.56% (-0.89 to -0.23) |
| Croatia | 0 (0 to 0) | 0.014 (0.012 to 0.017) | 0 (0 to 0) | 0.001 (0 to 0.001) | -0.96% (-0.98 to -0.95) | -9.37% (-10.47 to -8.25) |
| Cuba | 0.001 (0.001 to 0.001) | 0.01 (0.008 to 0.012) | 0.003 (0.002 to 0.004) | 0.062 (0.046 to 0.083) | 3.68% (2.2 to 5.67) | 7.38% (5.72 to 9.05) |
| Cyprus | 0 (0 to 0) | 0.011 (0.007 to 0.018) | 0 (0 to 0) | 0.001 (0.001 to 0.001) | -0.88% (-0.93 to -0.77) | -8.71% (-11.65 to -5.66) |
| Czechia | 0.001 (0.001 to 0.001) | 0.023 (0.019 to 0.028) | 0 (0 to 0) | 0.001 (0.001 to 0.001) | -0.97% (-0.98 to -0.96) | -10.51% (-11.83 to -9.17) |
| Democratic People's Republic of Korea | 0.025 (0.015 to 0.039) | 0.221 (0.132 to 0.345) | 0.003 (0.002 to 0.005) | 0.024 (0.014 to 0.038) | -0.87% (-0.93 to -0.77) | -8.11% (-8.38 to -7.83) |
| Democratic Republic of the Congo | 0.206 (0.142 to 0.281) | 1.306 (0.898 to 1.775) | 0.458 (0.328 to 0.599) | 1.2 (0.858 to 1.584) | 1.22% (0.49 to 2.37) | 0.61% (0.24 to 0.98) |
| Denmark | 0 (0 to 0) | 0.003 (0.002 to 0.003) | 0 (0 to 0) | 0.001 (0.001 to 0.002) | -0.49% (-0.64 to -0.29) | -4.54% (-6.16 to -2.88) |
| Djibouti | 0.002 (0.001 to 0.003) | 1.139 (0.743 to 1.626) | 0.006 (0.003 to 0.01) | 0.97 (0.502 to 1.554) | 1.82% (0.42 to 3.93) | -0.6% (-0.8 to -0.4) |
| Dominica | 0 (0 to 0) | 0.061 (0.045 to 0.083) | 0 (0 to 0) | 0.423 (0.287 to 0.599) | 5.56% (3.1 to 9.53) | 7.07% (5.3 to 8.86) |
| Dominican Republic | 0.001 (0.001 to 0.001) | 0.027 (0.021 to 0.035) | 0.004 (0.002 to 0.005) | 0.062 (0.041 to 0.091) | 2.27% (0.95 to 4.25) | 3.43% (2.9 to 3.97) |
| Ecuador | 0.001 (0 to 0.001) | 0.01 (0.009 to 0.013) | 0.006 (0.005 to 0.009) | 0.067 (0.048 to 0.096) | 10.54% (6.98 to 16.06) | 6.83% (5.35 to 8.34) |
| Egypt | 0.009 (0.007 to 0.011) | 0.033 (0.027 to 0.041) | 0.006 (0.004 to 0.009) | 0.012 (0.008 to 0.017) | -0.32% (-0.57 to 0.03) | -3.03% (-3.22 to -2.84) |
| El Salvador | 0.002 (0.002 to 0.003) | 0.077 (0.062 to 0.095) | 0.002 (0.001 to 0.003) | 0.045 (0.03 to 0.07) | -0.24% (-0.53 to 0.2) | -1.31% (-1.95 to -0.66) |
| Equatorial Guinea | 0.002 (0.002 to 0.003) | 1.279 (0.847 to 1.789) | 0.003 (0.002 to 0.005) | 0.479 (0.256 to 0.836) | 0.31% (-0.35 to 1.43) | -3.14% (-3.62 to -2.66) |
| Eritrea | 0.014 (0.009 to 0.02) | 1.098 (0.707 to 1.559) | 0.035 (0.023 to 0.052) | 1.085 (0.72 to 1.637) | 1.53% (0.55 to 3.18) | -0.16% (-0.74 to 0.42) |
| Estonia | 0 (0 to 0.001) | 0.06 (0.048 to 0.077) | 0 (0 to 0) | 0.001 (0.001 to 0.001) | -0.99% (-0.99 to -0.98) | -16.2% (-18.35 to -13.99) |
| Eswatini | 0.003 (0.002 to 0.004) | 0.94 (0.644 to 1.292) | 0.002 (0.001 to 0.003) | 0.25 (0.125 to 0.433) | -0.53% (-0.76 to -0.11) | -4.41% (-5.13 to -3.7) |
| Ethiopia | 0.238 (0.173 to 0.321) | 1.266 (0.921 to 1.713) | 0.283 (0.202 to 0.4) | 0.642 (0.455 to 0.906) | 0.19% (-0.27 to 0.87) | -2.72% (-2.9 to -2.54) |
| Fiji | 0.001 (0.001 to 0.002) | 0.297 (0.212 to 0.416) | 0.001 (0 to 0.001) | 0.159 (0.107 to 0.235) | -0.36% (-0.63 to 0.1) | -2.74% (-3.1 to -2.38) |
| Finland | 0 (0 to 0) | 0.006 (0.005 to 0.008) | 0 (0 to 0) | 0.001 (0.001 to 0.002) | -0.8% (-0.86 to -0.72) | -5.01% (-6.09 to -3.91) |
| France | 0.003 (0.002 to 0.003) | 0.009 (0.007 to 0.011) | 0.001 (0.001 to 0.001) | 0.004 (0.003 to 0.005) | -0.58% (-0.7 to -0.41) | -3.02% (-3.58 to -2.46) |
| Gabon | 0.005 (0.004 to 0.007) | 1.309 (0.969 to 1.725) | 0.005 (0.003 to 0.007) | 0.52 (0.32 to 0.792) | -0.07% (-0.43 to 0.48) | -3.34% (-3.87 to -2.81) |
| Gambia | 0.008 (0.005 to 0.011) | 1.954 (1.283 to 2.768) | 0.015 (0.01 to 0.021) | 1.47 (0.975 to 2.104) | 0.94% (0.22 to 2.25) | -1.08% (-1.5 to -0.66) |
| Georgia | 0 (0 to 0) | 0.007 (0.006 to 0.009) | 0 (0 to 0) | 0.009 (0.007 to 0.013) | -0.28% (-0.51 to 0.07) | 4.59% (3.04 to 6.16) |
| Germany | 0.002 (0.002 to 0.003) | 0.006 (0.005 to 0.007) | 0.001 (0 to 0.001) | 0.001 (0.001 to 0.002) | -0.78% (-0.84 to -0.7) | -5.55% (-6.08 to -5.02) |
| Ghana | 0.069 (0.047 to 0.091) | 1.002 (0.692 to 1.328) | 0.091 (0.059 to 0.124) | 0.518 (0.34 to 0.704) | 0.32% (-0.2 to 1.06) | -1.57% (-1.9 to -1.24) |
| Greece | 0 (0 to 0.001) | 0.009 (0.008 to 0.01) | 0 (0 to 0) | 0.004 (0.003 to 0.005) | -0.64% (-0.73 to -0.52) | -3.4% (-4.64 to -2.13) |
| Greenland | 0 (0 to 0) | 0.02 (0.013 to 0.031) | 0 (0 to 0) | 0.005 (0.003 to 0.007) | -0.81% (-0.9 to -0.65) | -7.24% (-8.53 to -5.92) |
| Grenada | 0 (0 to 0) | 0.049 (0.039 to 0.061) | 0 (0 to 0) | 0.07 (0.056 to 0.089) | 0.79% (0.28 to 1.49) | 2.6% (1.16 to 4.06) |
| Guam | 0 (0 to 0) | 0.005 (0.004 to 0.008) | 0 (0 to 0) | 0.036 (0.025 to 0.05) | 6.07% (3.16 to 10.95) | 7.97% (7.46 to 8.48) |
| Guatemala | 0.004 (0.003 to 0.005) | 0.1 (0.075 to 0.128) | 0.005 (0.003 to 0.007) | 0.046 (0.032 to 0.064) | 0.29% (-0.14 to 0.93) | -3.86% (-4.97 to -2.74) |
| Guinea | 0.057 (0.042 to 0.074) | 2.112 (1.562 to 2.754) | 0.087 (0.061 to 0.119) | 1.484 (1.038 to 2.031) | 0.54% (0.02 to 1.21) | -1.09% (-1.29 to -0.88) |
| Guinea-Bissau | 0.008 (0.006 to 0.011) | 1.768 (1.224 to 2.422) | 0.007 (0.005 to 0.011) | 0.757 (0.507 to 1.074) | -0.09% (-0.43 to 0.38) | -2.8% (-3.09 to -2.51) |
| Guyana | 0.001 (0 to 0.001) | 0.137 (0.1 to 0.178) | 0.002 (0.001 to 0.003) | 0.496 (0.331 to 0.712) | 2.64% (1.28 to 4.78) | 4.64% (3.32 to 5.98) |
| Haiti | 0.004 (0.003 to 0.006) | 0.145 (0.1 to 0.205) | 0.037 (0.024 to 0.052) | 0.518 (0.343 to 0.735) | 7.14% (4.36 to 11.24) | 5.51% (4.47 to 6.55) |
| Honduras | 0.006 (0.005 to 0.008) | 0.308 (0.235 to 0.399) | 0.002 (0.001 to 0.003) | 0.041 (0.024 to 0.064) | -0.66% (-0.81 to -0.46) | -7.32% (-7.64 to -6.99) |
| Hungary | 0.002 (0.002 to 0.003) | 0.043 (0.035 to 0.052) | 0 (0 to 0) | 0.002 (0.002 to 0.003) | -0.96% (-0.97 to -0.94) | -9.22% (-10.68 to -7.74) |
| Iceland | 0 (0 to 0) | 0.002 (0.001 to 0.002) | 0 (0 to 0) | 0.001 (0 to 0.001) | -0.66% (-0.76 to -0.52) | -3.94% (-4.37 to -3.51) |
| India | 1.831 (1.474 to 2.263) | 0.458 (0.369 to 0.564) | 1.301 (0.96 to 1.7) | 0.175 (0.13 to 0.23) | -0.29% (-0.5 to -0.03) | -3.66% (-3.95 to -3.36) |
| Indonesia | 0.184 (0.144 to 0.236) | 0.203 (0.158 to 0.26) | 0.077 (0.059 to 0.104) | 0.054 (0.041 to 0.073) | -0.58% (-0.71 to -0.37) | -4.92% (-5.46 to -4.39) |
| Iran (Islamic Republic of) | 0.018 (0.015 to 0.022) | 0.072 (0.058 to 0.087) | 0.005 (0.004 to 0.006) | 0.01 (0.009 to 0.013) | -0.72% (-0.78 to -0.62) | -6.93% (-7.38 to -6.48) |
| Iraq | 0.009 (0.006 to 0.013) | 0.13 (0.089 to 0.182) | 0.008 (0.005 to 0.012) | 0.039 (0.025 to 0.06) | -0.09% (-0.47 to 0.6) | -4.44% (-4.94 to -3.93) |
| Ireland | 0 (0 to 0) | 0.008 (0.006 to 0.01) | 0 (0 to 0) | 0.003 (0.003 to 0.005) | -0.41% (-0.61 to -0.11) | -2.1% (-3.88 to -0.3) |
| Israel | 0 (0 to 0) | 0.015 (0.013 to 0.018) | 0 (0 to 0) | 0.004 (0.003 to 0.004) | -0.59% (-0.7 to -0.46) | -5.51% (-6.67 to -4.34) |
| Italy | 0.003 (0.003 to 0.004) | 0.012 (0.01 to 0.014) | 0 (0 to 0.001) | 0.002 (0.002 to 0.002) | -0.86% (-0.9 to -0.82) | -5.88% (-6.35 to -5.4) |
| Jamaica | 0 (0 to 0) | 0.007 (0.006 to 0.009) | 0.001 (0.001 to 0.001) | 0.056 (0.038 to 0.081) | 8.96% (5.3 to 14.65) | 6.61% (4.06 to 9.22) |
| Japan | 0.008 (0.006 to 0.009) | 0.012 (0.01 to 0.015) | 0.001 (0.001 to 0.001) | 0.002 (0.002 to 0.002) | -0.87% (-0.91 to -0.83) | -6.38% (-6.66 to -6.09) |
| Jordan | 0.001 (0.001 to 0.001) | 0.048 (0.035 to 0.064) | 0 (0 to 0.001) | 0.006 (0.004 to 0.009) | -0.51% (-0.7 to -0.2) | -8.3% (-8.91 to -7.69) |
| Kazakhstan | 0.001 (0.001 to 0.001) | 0.012 (0.01 to 0.015) | 0 (0 to 0.001) | 0.005 (0.003 to 0.006) | -0.57% (-0.69 to -0.41) | -4.4% (-5.48 to -3.3) |
| Kenya | 0.072 (0.054 to 0.094) | 0.858 (0.641 to 1.124) | 0.123 (0.084 to 0.171) | 0.525 (0.358 to 0.727) | 0.7% (0.18 to 1.52) | -1.45% (-1.98 to -0.92) |
| Kiribati | 0 (0 to 0) | 0.87 (0.606 to 1.2) | 0.001 (0.001 to 0.001) | 1.42 (0.977 to 1.957) | 1.67% (0.67 to 3.21) | 1.85% (1.1 to 2.61) |
| Kuwait | 0 (0 to 0) | 0.006 (0.005 to 0.007) | 0 (0 to 0) | 0.002 (0.001 to 0.003) | 0.28% (-0.14 to 0.88) | -4.89% (-6.38 to -3.37) |
| Kyrgyzstan | 0 (0 to 0.001) | 0.022 (0.018 to 0.026) | 0 (0 to 0) | 0.01 (0.008 to 0.013) | -0.26% (-0.45 to -0.01) | -1.13% (-1.79 to -0.47) |
| Lao People's Democratic Republic | 0.002 (0.001 to 0.003) | 0.089 (0.062 to 0.131) | 0.001 (0.001 to 0.002) | 0.036 (0.024 to 0.051) | -0.17% (-0.5 to 0.27) | -3.51% (-3.89 to -3.13) |
| Latvia | 0 (0 to 0) | 0.015 (0.012 to 0.018) | 0 (0 to 0) | 0.004 (0.002 to 0.006) | -0.85% (-0.91 to -0.75) | -4.82% (-6.34 to -3.27) |
| Lebanon | 0.001 (0.001 to 0.001) | 0.052 (0.035 to 0.073) | 0 (0 to 0) | 0.011 (0.008 to 0.016) | -0.62% (-0.77 to -0.36) | -5.35% (-5.62 to -5.08) |
| Lesotho | 0.008 (0.005 to 0.011) | 1.013 (0.676 to 1.407) | 0.01 (0.006 to 0.015) | 0.884 (0.498 to 1.372) | 0.25% (-0.33 to 1.2) | 1.36% (0.55 to 2.18) |
| Liberia | 0.016 (0.012 to 0.022) | 2.061 (1.506 to 2.737) | 0.029 (0.02 to 0.04) | 1.249 (0.847 to 1.722) | 0.76% (0.11 to 1.68) | -1.25% (-1.57 to -0.92) |
| Libya | 0.001 (0 to 0.001) | 0.046 (0.032 to 0.065) | 0 (0 to 0.001) | 0.01 (0.006 to 0.014) | -0.43% (-0.68 to -0.03) | -4.48% (-5.21 to -3.75) |
| Lithuania | 0 (0 to 0.001) | 0.024 (0.019 to 0.029) | 0 (0 to 0) | 0.002 (0.001 to 0.003) | -0.95% (-0.97 to -0.93) | -8.48% (-10.06 to -6.87) |
| Luxembourg | 0 (0 to 0) | 0.009 (0.008 to 0.011) | 0 (0 to 0) | 0.003 (0.002 to 0.004) | -0.5% (-0.64 to -0.31) | -3.87% (-4.17 to -3.56) |
| Madagascar | 0.081 (0.063 to 0.101) | 1.664 (1.297 to 2.1) | 0.133 (0.096 to 0.18) | 1.097 (0.777 to 1.518) | 0.64% (0.12 to 1.32) | -1.77% (-2.17 to -1.37) |
| Malawi | 0.062 (0.045 to 0.081) | 1.616 (1.182 to 2.13) | 0.057 (0.036 to 0.083) | 0.694 (0.438 to 1.016) | -0.07% (-0.45 to 0.48) | -2.78% (-3.2 to -2.36) |
| Malaysia | 0.01 (0.007 to 0.013) | 0.106 (0.081 to 0.138) | 0.008 (0.005 to 0.011) | 0.045 (0.03 to 0.065) | -0.2% (-0.51 to 0.31) | -3.18% (-3.61 to -2.74) |
| Maldives | 0 (0 to 0) | 0.104 (0.075 to 0.144) | 0 (0 to 0) | 0.015 (0.011 to 0.02) | -0.64% (-0.76 to -0.43) | -6.74% (-7.39 to -6.1) |
| Mali | 0.053 (0.042 to 0.069) | 1.424 (1.104 to 1.83) | 0.084 (0.056 to 0.119) | 0.865 (0.574 to 1.221) | 0.57% (0.02 to 1.29) | -2.19% (-2.47 to -1.9) |
| Malta | 0 (0 to 0) | 0.022 (0.017 to 0.027) | 0 (0 to 0) | 0.009 (0.007 to 0.012) | -0.57% (-0.69 to -0.41) | -2.46% (-2.8 to -2.11) |
| Marshall Islands | 0 (0 to 0) | 0.271 (0.194 to 0.359) | 0 (0 to 0) | 0.195 (0.114 to 0.297) | 0.11% (-0.4 to 0.82) | -1.36% (-1.65 to -1.07) |
| Mauritania | 0.029 (0.022 to 0.037) | 3.464 (2.69 to 4.493) | 0.035 (0.022 to 0.052) | 1.926 (1.209 to 2.835) | 0.23% (-0.25 to 0.9) | -1.47% (-1.69 to -1.24) |
| Mauritius | 0 (0 to 0) | 0.027 (0.022 to 0.034) | 0 (0 to 0) | 0.033 (0.023 to 0.044) | 0.19% (-0.21 to 0.75) | 1.26% (-0.18 to 2.71) |
| Mexico | 0.041 (0.035 to 0.049) | 0.093 (0.079 to 0.109) | 0.022 (0.016 to 0.029) | 0.032 (0.024 to 0.042) | -0.47% (-0.62 to -0.27) | -3.86% (-4.32 to -3.4) |
| Micronesia (Federated States of) | 0 (0 to 0) | 0.35 (0.229 to 0.51) | 0 (0 to 0) | 0.173 (0.058 to 0.284) | -0.44% (-0.8 to -0.01) | -2.85% (-3 to -2.7) |
| Monaco | 0 (0 to 0) | 0.002 (0.001 to 0.003) | 0 (0 to 0) | 0.001 (0 to 0.001) | -0.63% (-0.78 to -0.32) | -3.3% (-3.65 to -2.94) |
| Mongolia | 0.001 (0 to 0.001) | 0.05 (0.036 to 0.067) | 0 (0 to 0.001) | 0.02 (0.013 to 0.03) | -0.31% (-0.59 to 0.13) | -3.4% (-3.87 to -2.93) |
| Montenegro | 0 (0 to 0) | 0.002 (0.001 to 0.002) | 0 (0 to 0) | 0.001 (0.001 to 0.002) | -0.39% (-0.64 to 0.06) | -1.19% (-1.78 to -0.59) |
| Morocco | 0.01 (0.007 to 0.013) | 0.076 (0.057 to 0.1) | 0.005 (0.004 to 0.008) | 0.028 (0.019 to 0.043) | -0.44% (-0.64 to -0.08) | -3.77% (-4.23 to -3.32) |
| Mozambique | 0.048 (0.034 to 0.065) | 0.856 (0.613 to 1.167) | 0.107 (0.065 to 0.155) | 0.837 (0.503 to 1.211) | 1.23% (0.31 to 2.47) | 1.19% (0.64 to 1.74) |
| Myanmar | 0.019 (0.013 to 0.029) | 0.088 (0.059 to 0.13) | 0.043 (0.028 to 0.063) | 0.144 (0.094 to 0.21) | 1.25% (0.34 to 2.79) | 1.61% (1.27 to 1.94) |
| Namibia | 0.005 (0.003 to 0.007) | 0.866 (0.527 to 1.198) | 0.005 (0.003 to 0.009) | 0.42 (0.234 to 0.687) | 0.02% (-0.45 to 1.11) | -2.17% (-2.52 to -1.81) |
| Nauru | 0 (0 to 0) | 0.432 (0.287 to 0.637) | 0 (0 to 0) | 0.229 (0.14 to 0.371) | -0.39% (-0.65 to 0.05) | -2.43% (-2.79 to -2.06) |
| Nepal | 0.007 (0.005 to 0.009) | 0.07 (0.052 to 0.094) | 0.002 (0.001 to 0.003) | 0.012 (0.008 to 0.017) | -0.67% (-0.8 to -0.48) | -6.32% (-6.88 to -5.75) |
| Netherlands | 0.001 (0.001 to 0.001) | 0.009 (0.007 to 0.011) | 0 (0 to 0) | 0.002 (0.001 to 0.002) | -0.84% (-0.88 to -0.78) | -7.3% (-8.04 to -6.56) |
| New Zealand | 0 (0 to 0) | 0.005 (0.004 to 0.007) | 0 (0 to 0) | 0.003 (0.002 to 0.004) | -0.37% (-0.54 to -0.13) | -1.81% (-2.55 to -1.06) |
| Nicaragua | 0 (0 to 0) | 0.01 (0.008 to 0.012) | 0 (0 to 0) | 0.005 (0.003 to 0.007) | -0.02% (-0.36 to 0.43) | -1.6% (-2.53 to -0.65) |
| Niger | 0.044 (0.031 to 0.057) | 1.37 (0.974 to 1.818) | 0.118 (0.075 to 0.166) | 1.354 (0.87 to 1.907) | 1.72% (0.71 to 3.06) | -0.34% (-0.58 to -0.1) |
| Nigeria | 0.416 (0.259 to 0.646) | 1.095 (0.686 to 1.706) | 0.71 (0.43 to 1.17) | 0.691 (0.419 to 1.137) | 0.71% (-0.03 to 2.01) | -1.79% (-2.32 to -1.25) |
| Niue | 0 (0 to 0) | 0.181 (0.116 to 0.276) | 0 (0 to 0) | 0.082 (0.041 to 0.133) | -0.65% (-0.83 to -0.36) | -3.35% (-3.55 to -3.15) |
| North Macedonia | 0 (0 to 0) | 0.014 (0.01 to 0.018) | 0 (0 to 0) | 0.003 (0.002 to 0.004) | -0.8% (-0.88 to -0.67) | -6.41% (-6.96 to -5.86) |
| Northern Mariana Islands | 0 (0 to 0) | 0.148 (0.097 to 0.214) | 0 (0 to 0) | 0.146 (0.098 to 0.209) | -0.26% (-0.58 to 0.3) | 0.03% (-0.69 to 0.75) |
| Norway | 0 (0 to 0) | 0.006 (0.005 to 0.008) | 0 (0 to 0) | 0.001 (0.001 to 0.001) | -0.87% (-0.9 to -0.83) | -7.45% (-9.2 to -5.67) |
| Oman | 0 (0 to 0.001) | 0.055 (0.037 to 0.08) | 0 (0 to 0) | 0.009 (0.006 to 0.012) | -0.5% (-0.69 to -0.18) | -6.45% (-6.82 to -6.07) |
| Pakistan | 0.192 (0.148 to 0.246) | 0.407 (0.314 to 0.518) | 0.444 (0.309 to 0.601) | 0.386 (0.266 to 0.525) | 1.31% (0.53 to 2.34) | -0.36% (-0.8 to 0.08) |
| Palau | 0 (0 to 0) | 0.035 (0.022 to 0.053) | 0 (0 to 0) | 0.019 (0.012 to 0.03) | -0.57% (-0.76 to -0.22) | -1.97% (-2.05 to -1.9) |
| Palestine | 0 (0 to 0.001) | 0.057 (0.039 to 0.084) | 0 (0 to 0) | 0.01 (0.007 to 0.013) | -0.5% (-0.7 to -0.18) | -5.58% (-5.98 to -5.19) |
| Panama | 0.001 (0.001 to 0.001) | 0.05 (0.041 to 0.06) | 0.001 (0.001 to 0.002) | 0.072 (0.049 to 0.101) | 1.37% (0.57 to 2.44) | 1.89% (1.4 to 2.38) |
| Papua New Guinea | 0.004 (0.002 to 0.005) | 0.197 (0.138 to 0.265) | 0.009 (0.006 to 0.012) | 0.174 (0.123 to 0.248) | 1.35% (0.54 to 2.64) | -0.54% (-0.84 to -0.23) |
| Paraguay | 0 (0 to 0) | 0.011 (0.008 to 0.014) | 0.001 (0.001 to 0.002) | 0.034 (0.022 to 0.049) | 5.05% (2.78 to 8.5) | 3.62% (1.88 to 5.39) |
| Peru | 0.006 (0.005 to 0.007) | 0.052 (0.041 to 0.066) | 0.011 (0.007 to 0.016) | 0.06 (0.039 to 0.09) | 0.88% (0.19 to 1.97) | 0.13% (-1.05 to 1.32) |
| Philippines | 0.06 (0.048 to 0.074) | 0.19 (0.151 to 0.234) | 0.065 (0.045 to 0.092) | 0.112 (0.077 to 0.158) | 0.08% (-0.28 to 0.59) | -1.52% (-1.64 to -1.39) |
| Poland | 0.005 (0.004 to 0.006) | 0.027 (0.023 to 0.032) | 0 (0 to 0) | 0.001 (0.001 to 0.002) | -0.95% (-0.97 to -0.93) | -11.06% (-12.29 to -9.81) |
| Portugal | 0.001 (0.001 to 0.001) | 0.017 (0.014 to 0.021) | 0 (0 to 0) | 0.002 (0.001 to 0.002) | -0.91% (-0.93 to -0.87) | -8.95% (-10.17 to -7.71) |
| Puerto Rico | 0 (0 to 0.001) | 0.024 (0.02 to 0.029) | 0 (0 to 0) | 0.007 (0.005 to 0.01) | -0.73% (-0.82 to -0.6) | -4.49% (-5.54 to -3.43) |
| Qatar | 0 (0 to 0) | 0.05 (0.034 to 0.068) | 0 (0 to 0) | 0.007 (0.004 to 0.009) | -0.24% (-0.55 to 0.3) | -6.86% (-7.29 to -6.44) |
| Republic of Korea | 0.002 (0.002 to 0.002) | 0.007 (0.006 to 0.009) | 0.001 (0 to 0.001) | 0.002 (0.002 to 0.003) | -0.73% (-0.82 to -0.6) | -4.86% (-5.47 to -4.25) |
| Republic of Moldova | 0 (0 to 0) | 0.012 (0.01 to 0.015) | 0 (0 to 0) | 0.004 (0.003 to 0.005) | -0.78% (-0.84 to -0.69) | -4.02% (-4.69 to -3.35) |
| Romania | 0 (0 to 0.001) | 0.004 (0.003 to 0.006) | 0 (0 to 0.001) | 0.006 (0.004 to 0.007) | -0.09% (-0.39 to 0.35) | 0.49% (-0.6 to 1.6) |
| Russian Federation | 0.107 (0.091 to 0.125) | 0.134 (0.114 to 0.157) | 0.008 (0.005 to 0.01) | 0.01 (0.007 to 0.014) | -0.93% (-0.95 to -0.9) | -9.79% (-10.27 to -9.3) |
| Rwanda | 0.018 (0.014 to 0.025) | 0.663 (0.492 to 0.906) | 0.016 (0.01 to 0.023) | 0.259 (0.167 to 0.378) | -0.15% (-0.52 to 0.37) | -3.35% (-3.75 to -2.95) |
| Saint Kitts and Nevis | 0 (0 to 0) | 0.016 (0.013 to 0.02) | 0 (0 to 0) | 0.023 (0.007 to 0.038) | 0.97% (-0.43 to 2.66) | 1.83% (1.1 to 2.55) |
| Saint Lucia | 0 (0 to 0) | 0.012 (0.01 to 0.015) | 0 (0 to 0) | 0.015 (0.011 to 0.019) | 0.56% (0.07 to 1.19) | 1.75% (1.21 to 2.29) |
| Saint Vincent and the Grenadines | 0 (0 to 0) | 0.178 (0.145 to 0.214) | 0 (0 to 0) | 0.245 (0.187 to 0.313) | 0.4% (0 to 0.95) | 1.3% (0.98 to 1.62) |
| Samoa | 0 (0 to 0) | 0.101 (0.064 to 0.152) | 0 (0 to 0) | 0.039 (0.017 to 0.063) | -0.47% (-0.79 to 0.01) | -4.2% (-4.7 to -3.69) |
| San Marino | 0 (0 to 0) | 0.003 (0.002 to 0.005) | 0 (0 to 0) | 0.002 (0.001 to 0.003) | -0.17% (-0.61 to 0.65) | -1.37% (-1.49 to -1.25) |
| Sao Tome and Principe | 0 (0 to 0.001) | 0.786 (0.417 to 1.156) | 0 (0 to 0) | 0.313 (0.212 to 0.449) | -0.1% (-0.48 to 0.86) | -4.11% (-4.84 to -3.37) |
| Saudi Arabia | 0.003 (0.002 to 0.004) | 0.051 (0.033 to 0.074) | 0.005 (0.003 to 0.007) | 0.022 (0.014 to 0.032) | 0.48% (-0.15 to 1.53) | -2.05% (-2.51 to -1.59) |
| Senegal | 0.054 (0.04 to 0.071) | 1.721 (1.263 to 2.262) | 0.108 (0.072 to 0.15) | 1.547 (1.027 to 2.159) | 1% (0.24 to 2.03) | 0.22% (-0.04 to 0.48) |
| Serbia | 0 (0 to 0) | 0.005 (0.004 to 0.007) | 0 (0 to 0) | 0.002 (0.001 to 0.002) | -0.72% (-0.83 to -0.55) | -3.71% (-4.09 to -3.34) |
| Seychelles | 0 (0 to 0) | 0.121 (0.093 to 0.153) | 0 (0 to 0) | 0.058 (0.042 to 0.077) | -0.38% (-0.58 to -0.14) | -1.84% (-2.29 to -1.39) |
| Sierra Leone | 0.021 (0.014 to 0.029) | 1.289 (0.883 to 1.787) | 0.061 (0.041 to 0.084) | 1.495 (1.009 to 2.049) | 1.89% (0.79 to 3.46) | 1.09% (0.74 to 1.44) |
| Singapore | 0 (0 to 0.001) | 0.021 (0.017 to 0.026) | 0 (0 to 0) | 0.002 (0.001 to 0.002) | -0.87% (-0.91 to -0.82) | -8.11% (-9.38 to -6.83) |
| Slovakia | 0 (0 to 0) | 0.01 (0.007 to 0.013) | 0 (0 to 0) | 0.002 (0.002 to 0.003) | -0.77% (-0.86 to -0.64) | -4.14% (-5.25 to -3.01) |
| Slovenia | 0 (0 to 0) | 0.023 (0.015 to 0.032) | 0 (0 to 0) | 0.002 (0.001 to 0.002) | -0.94% (-0.97 to -0.9) | -6.82% (-8.84 to -4.76) |
| Solomon Islands | 0.001 (0.001 to 0.001) | 0.618 (0.397 to 0.939) | 0.002 (0.001 to 0.003) | 0.607 (0.378 to 0.881) | 1.24% (0.33 to 2.8) | -0.1% (-0.35 to 0.15) |
| Somalia | 0.032 (0.019 to 0.049) | 1.162 (0.681 to 1.771) | 0.085 (0.055 to 0.125) | 1.088 (0.687 to 1.607) | 1.64% (0.71 to 3.22) | 0.03% (-0.14 to 0.21) |
| South Africa | 0.185 (0.149 to 0.226) | 0.954 (0.77 to 1.156) | 0.071 (0.043 to 0.107) | 0.22 (0.136 to 0.327) | -0.62% (-0.77 to -0.42) | -4.13% (-5.56 to -2.68) |
| South Sudan | 0.014 (0.009 to 0.021) | 0.647 (0.404 to 0.969) | 0.02 (0.011 to 0.034) | 0.505 (0.28 to 0.861) | 0.45% (-0.15 to 1.53) | -0.5% (-0.77 to -0.23) |
| Spain | 0.001 (0.001 to 0.001) | 0.005 (0.004 to 0.006) | 0 (0 to 0) | 0.001 (0.001 to 0.002) | -0.73% (-0.8 to -0.62) | -3.9% (-4.67 to -3.12) |
| Sri Lanka | 0.028 (0.022 to 0.036) | 0.294 (0.225 to 0.376) | 0.021 (0.014 to 0.03) | 0.191 (0.127 to 0.276) | -0.26% (-0.53 to 0.17) | -1.42% (-1.6 to -1.23) |
| Sudan | 0.007 (0.005 to 0.009) | 0.072 (0.051 to 0.097) | 0.006 (0.004 to 0.01) | 0.03 (0.017 to 0.046) | -0.06% (-0.48 to 0.61) | -2.55% (-2.93 to -2.18) |
| Suriname | 0 (0 to 0.001) | 0.215 (0.136 to 0.286) | 0.001 (0.001 to 0.001) | 0.317 (0.223 to 0.433) | 1.15% (0.37 to 2.58) | 1.63% (1.2 to 2.06) |
| Sweden | 0 (0 to 0) | 0.004 (0.003 to 0.005) | 0 (0 to 0) | 0.001 (0.001 to 0.002) | -0.66% (-0.75 to -0.52) | -4.16% (-5.31 to -2.99) |
| Switzerland | 0 (0 to 0) | 0.004 (0.003 to 0.005) | 0 (0 to 0) | 0.002 (0.001 to 0.002) | -0.52% (-0.66 to -0.32) | -4.07% (-5.2 to -2.93) |
| Syrian Arab Republic | 0.002 (0.001 to 0.003) | 0.041 (0.028 to 0.056) | 0 (0 to 0.001) | 0.005 (0.003 to 0.008) | -0.81% (-0.89 to -0.69) | -7.21% (-7.78 to -6.63) |
| Taiwan (Province of China) | 0.003 (0.002 to 0.003) | 0.023 (0.019 to 0.029) | 0.001 (0 to 0.001) | 0.005 (0.003 to 0.006) | -0.79% (-0.86 to -0.69) | -5.7% (-6.43 to -4.96) |
| Tajikistan | 0.001 (0.001 to 0.002) | 0.052 (0.04 to 0.067) | 0 (0 to 0.001) | 0.009 (0.006 to 0.013) | -0.66% (-0.78 to -0.47) | -7.36% (-8.13 to -6.58) |
| Thailand | 0.007 (0.005 to 0.01) | 0.021 (0.015 to 0.029) | 0.007 (0.004 to 0.01) | 0.021 (0.013 to 0.031) | -0.01% (-0.42 to 0.65) | -0.31% (-1.25 to 0.64) |
| Timor-Leste | 0.001 (0.001 to 0.001) | 0.27 (0.164 to 0.383) | 0.001 (0 to 0.002) | 0.244 (0.052 to 0.364) | 0.45% (-0.65 to 1.72) | -1.08% (-1.73 to -0.43) |
| Togo | 0.024 (0.018 to 0.031) | 1.546 (1.139 to 2.033) | 0.025 (0.016 to 0.036) | 0.644 (0.407 to 0.915) | 0.07% (-0.34 to 0.62) | -3.2% (-3.66 to -2.73) |
| Tokelau | 0 (0 to 0) | 0.277 (0.169 to 0.424) | 0 (0 to 0) | 0.135 (0.078 to 0.214) | -0.56% (-0.76 to -0.19) | -2.7% (-2.8 to -2.6) |
| Tonga | 0 (0 to 0) | 0.23 (0.169 to 0.305) | 0 (0 to 0) | 0.144 (0.09 to 0.214) | -0.23% (-0.55 to 0.24) | -2.04% (-2.22 to -1.85) |
| Trinidad and Tobago | 0 (0 to 0) | 0.021 (0.016 to 0.026) | 0.001 (0 to 0.001) | 0.08 (0.051 to 0.121) | 3.41% (1.65 to 6.09) | 5.83% (4.37 to 7.31) |
| Tunisia | 0.001 (0.001 to 0.002) | 0.031 (0.023 to 0.04) | 0.001 (0 to 0.001) | 0.009 (0.006 to 0.014) | -0.56% (-0.74 to -0.26) | -4.15% (-4.3 to -3.99) |
| Turkey | 0.011 (0.009 to 0.015) | 0.038 (0.028 to 0.05) | 0.002 (0.001 to 0.003) | 0.004 (0.003 to 0.006) | -0.83% (-0.89 to -0.73) | -8.93% (-9.97 to -7.88) |
| Turkmenistan | 0.001 (0.001 to 0.001) | 0.042 (0.033 to 0.052) | 0.001 (0.001 to 0.001) | 0.037 (0.025 to 0.051) | 0.27% (-0.14 to 0.87) | -0.05% (-0.54 to 0.44) |
| Tuvalu | 0 (0 to 0) | 0.339 (0.231 to 0.481) | 0 (0 to 0) | 0.139 (0.083 to 0.21) | -0.55% (-0.75 to -0.22) | -3.35% (-3.48 to -3.22) |
| Uganda | 0.03 (0.019 to 0.044) | 0.46 (0.284 to 0.671) | 0.098 (0.064 to 0.143) | 0.559 (0.368 to 0.813) | 2.28% (0.91 to 4.59) | 0.76% (0.12 to 1.41) |
| Ukraine | 0.002 (0.002 to 0.003) | 0.01 (0.007 to 0.012) | 0.001 (0 to 0.001) | 0.003 (0.002 to 0.005) | -0.74% (-0.83 to -0.61) | -4.14% (-4.97 to -3.3) |
| United Arab Emirates | 0 (0 to 0) | 0.036 (0.024 to 0.052) | 0 (0 to 0) | 0.007 (0.004 to 0.01) | 0.03% (-0.44 to 0.83) | -6.32% (-6.86 to -5.78) |
| United Kingdom | 0.006 (0.005 to 0.007) | 0.019 (0.016 to 0.023) | 0.001 (0.001 to 0.001) | 0.004 (0.003 to 0.004) | -0.79% (-0.84 to -0.73) | -6.15% (-6.71 to -5.59) |
| United Republic of Tanzania | 0.168 (0.121 to 0.225) | 1.726 (1.228 to 2.337) | 0.25 (0.168 to 0.348) | 1.012 (0.673 to 1.428) | 0.49% (-0.04 to 1.34) | -1.09% (-1.48 to -0.71) |
| United States of America | 0.041 (0.034 to 0.049) | 0.03 (0.025 to 0.036) | 0.019 (0.016 to 0.023) | 0.013 (0.011 to 0.016) | -0.53% (-0.64 to -0.4) | -2.35% (-2.67 to -2.03) |
| United States Virgin Islands | 0 (0 to 0) | 0.015 (0.011 to 0.02) | 0 (0 to 0) | 0.011 (0.007 to 0.018) | -0.43% (-0.67 to -0.02) | -0.5% (-1.21 to 0.22) |
| Uruguay | 0 (0 to 0) | 0.015 (0.012 to 0.018) | 0 (0 to 0) | 0.005 (0.004 to 0.006) | -0.65% (-0.73 to -0.54) | -5.65% (-6.49 to -4.8) |
| Uzbekistan | 0.001 (0.001 to 0.001) | 0.012 (0.009 to 0.014) | 0.001 (0.001 to 0.002) | 0.008 (0.006 to 0.01) | 0.19% (-0.16 to 0.68) | -0.44% (-1.24 to 0.36) |
| Vanuatu | 0 (0 to 0) | 0.139 (0.079 to 0.221) | 0 (0 to 0) | 0.121 (0.06 to 0.187) | 0.85% (-0.16 to 2.46) | -1.27% (-1.6 to -0.95) |
| Venezuela (Bolivarian Republic of) | 0.001 (0.001 to 0.001) | 0.011 (0.009 to 0.013) | 0.01 (0.007 to 0.014) | 0.068 (0.047 to 0.096) | 7.84% (4.9 to 12.13) | 6.44% (5.41 to 7.47) |
| Viet Nam | 0.004 (0.003 to 0.007) | 0.012 (0.007 to 0.018) | 0.002 (0.001 to 0.003) | 0.003 (0.002 to 0.005) | -0.59% (-0.77 to -0.28) | -3.52% (-3.88 to -3.17) |
| Yemen | 0.005 (0.003 to 0.008) | 0.098 (0.052 to 0.151) | 0.015 (0.008 to 0.024) | 0.097 (0.054 to 0.146) | 1.88% (0.43 to 4.83) | -0.04% (-0.5 to 0.42) |
| Zambia | 0.033 (0.024 to 0.043) | 1.131 (0.83 to 1.517) | 0.037 (0.024 to 0.053) | 0.478 (0.304 to 0.699) | 0.12% (-0.31 to 0.7) | -3.31% (-3.58 to -3.05) |
| Zimbabwe | 0.053 (0.04 to 0.068) | 1.222 (0.91 to 1.576) | 0.074 (0.05 to 0.103) | 0.954 (0.643 to 1.323) | 0.4% (-0.1 to 1.13) | 1.44% (0.29 to 2.6) |
