# Supplementary figures and images for: Global burden and trends of ectopic pregnancy: An observational trend study from 1990 to 2019
Source: PLoS One. 2023 Oct 26;18(10):e0291316. doi: 10.1371/journal.pone.0291316 (PMC10602312; doi:10.1371/journal.pone.0291316)

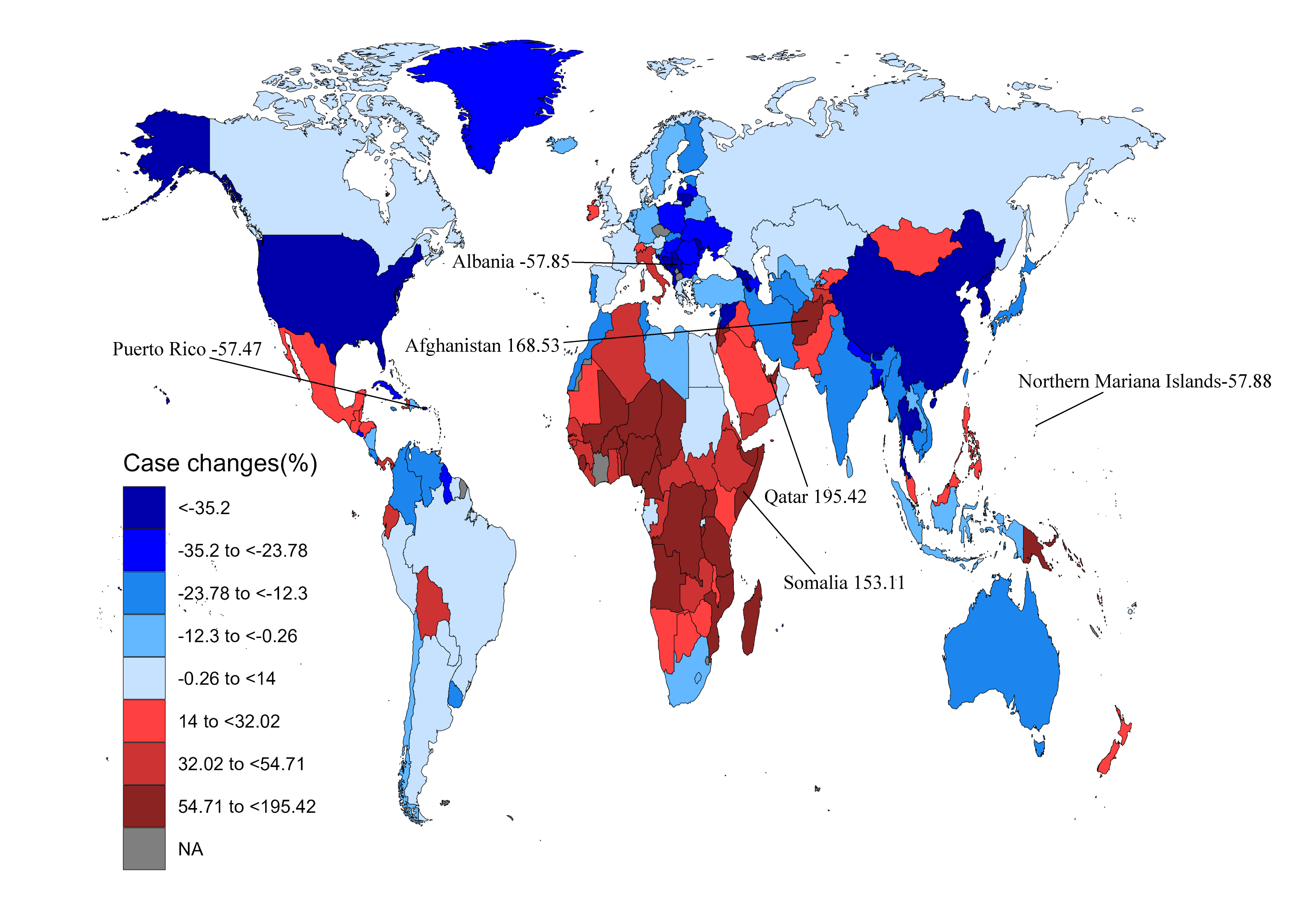

Supplement: S1 Fig — (TIF) [file pone.0291316.s004.tif]

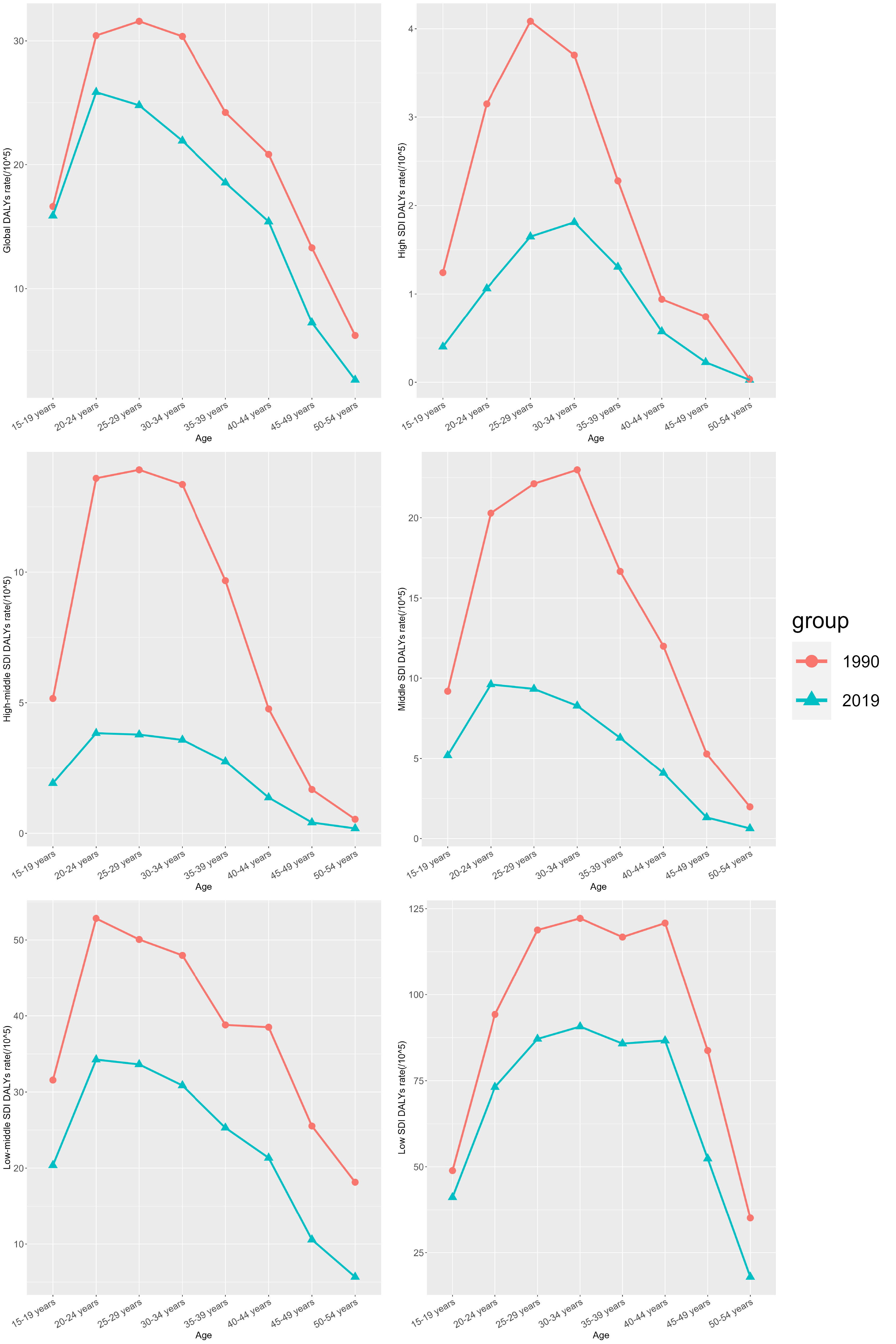

Supplement: S2 Fig — (TIF) [file pone.0291316.s005.tif]

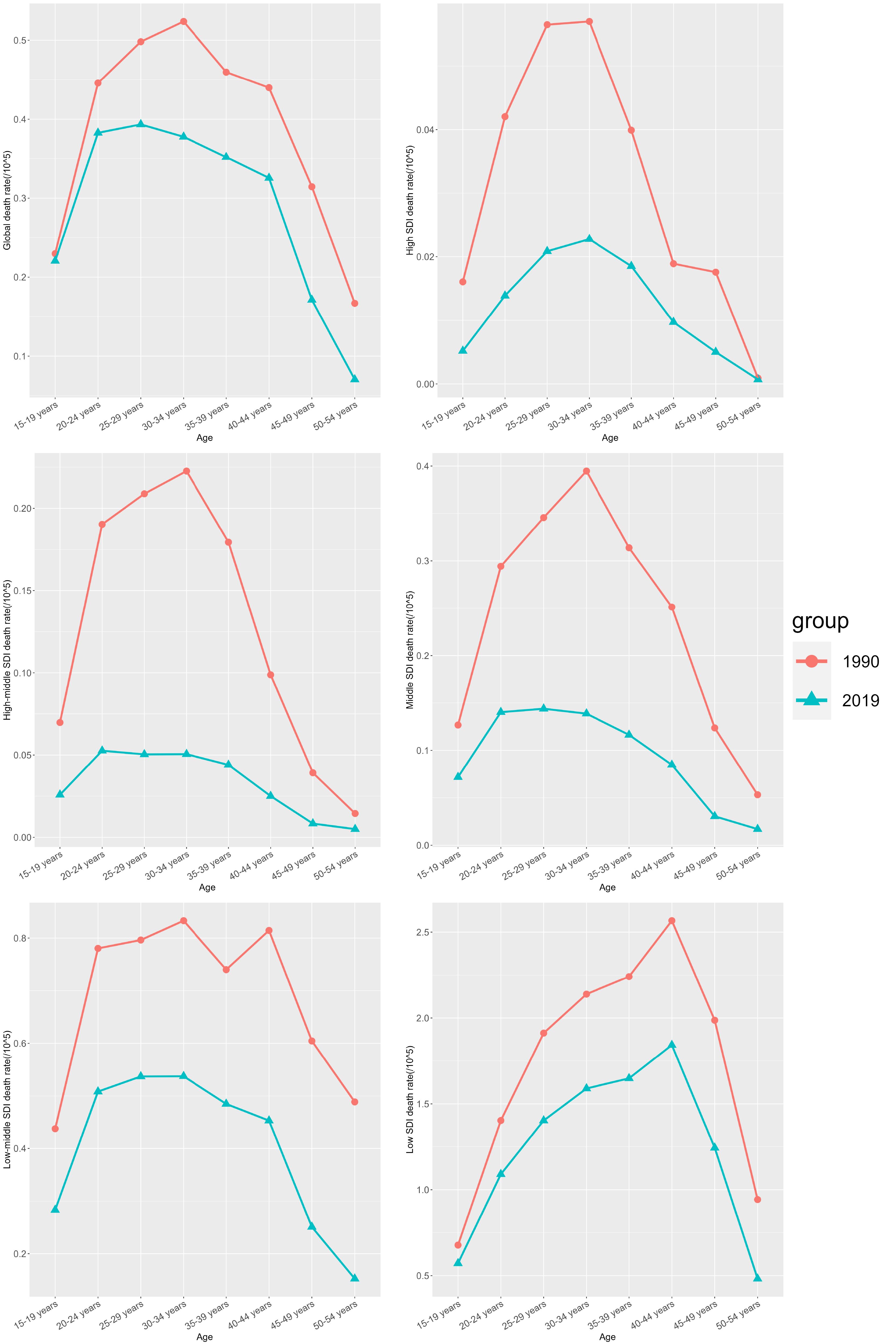

Supplement: S3 Fig — (TIF) [file pone.0291316.s006.tif]

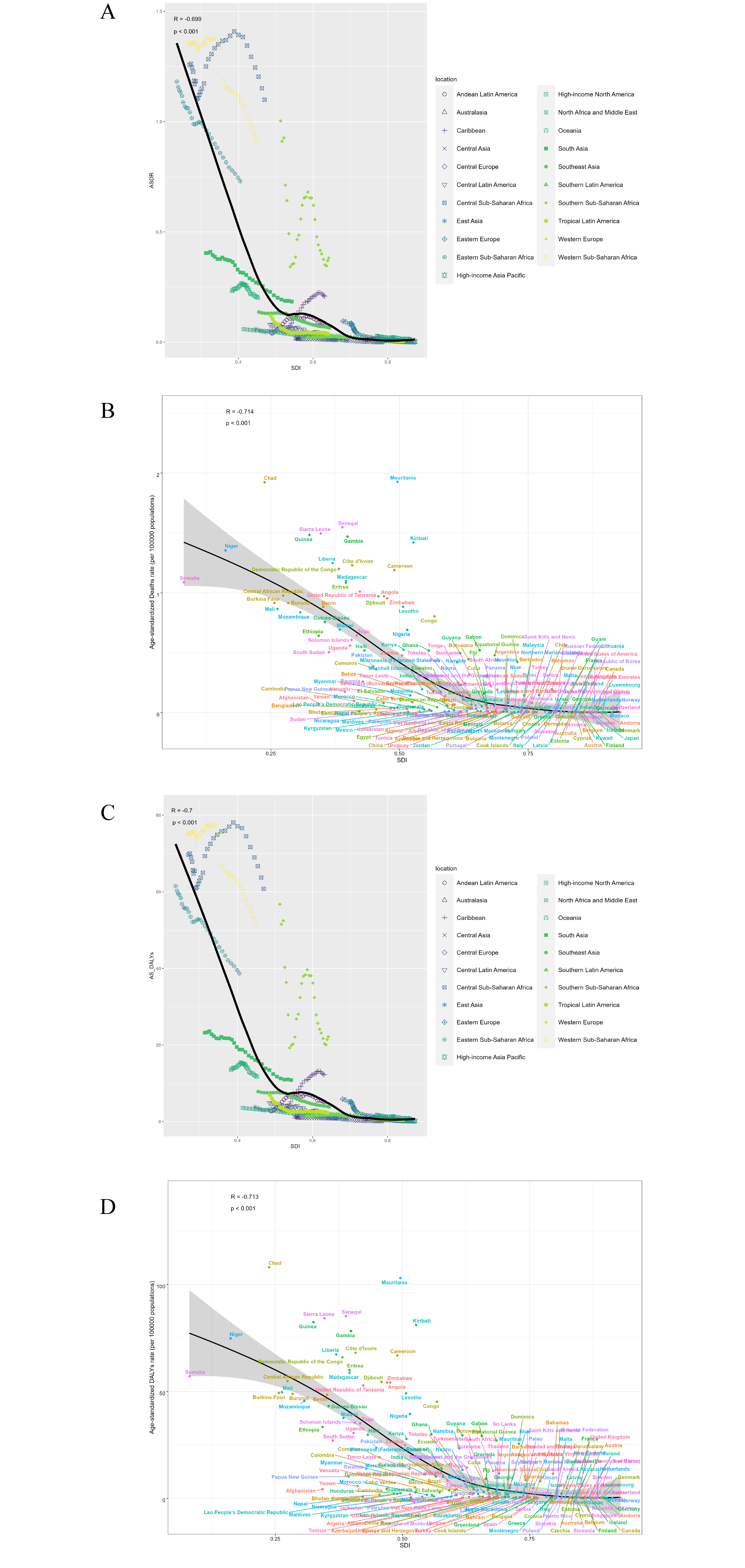

Supplement: S4 Fig — (A) ASDR for EP for different regions and (B) countries and territories by SDI, 1990–2019; (C)AS-DALYs for EP for different regions and (D) countries and territories by SDI, 1990–2019. (TIF) [file pone.0291316.s007.tif]
