# Supplementary material for: Global burden and trends of ectopic pregnancy: An observational trend study from 1990 to 2019
Source: PLoS One. 2023 Oct 26;18(10):e0291316. doi: 10.1371/journal.pone.0291316 (PMC10602312; doi:10.1371/journal.pone.0291316)
Supplement: Attachment — Submitted filename: Comments and Suggestions.docx [file pone.0291316.s008.docx]

I appreciate the authors for this intensive research work on the topic “Global burden and trends of ectopic pregnancy: An observational trend study from 1990 to 2019”. The presentation of the data seems strong, and the paper is generally well-written, with an appropriate focus. However, this study may be accepted for publication with the following suggestions and comments for further improvements.

**Abstract**

Comment 1: The abstract is written with an appropriate focus.

**Introduction**

Comment 2: The introduction section is poorly written.

Comment 3: In addition to the current contents of the introduction section, add at least two paragraphs in the social context like.

1. What is the situation of EP in developed, developing, and underdeveloped countries? elaborate.

2. How is it changing over time?

3. Where it is still high from the previous literature.

4. Elaborate if it is declining, then why is your study important?

**Material and Methods**

Comment 4: Written Properly.

**Results and Discussion**

Comment 5: Figure resolution is very poor. Please provide high-resolution figures.

Comment 6: Add some discussion for underdeveloped and developing countries’ situations.

Comment 7: The conclusion is written very well.
