# Supplementary material for: Global burden and trends of ectopic pregnancy: An observational trend study from 1990 to 2019
Source: PLoS One. 2023 Oct 26;18(10):e0291316. doi: 10.1371/journal.pone.0291316 (PMC10602312; doi:10.1371/journal.pone.0291316)
Supplement: Attachment — Submitted filename: Response to Reviewers.docx [file pone.0291316.s009.docx]

Dear Editor,

Thank you for your letter and for the reviewers' comments concerning our manuscript entitled " Global burden and trends of ectopic pregnancy: An observational trend study from 1990 to 2019" (PONE-D-22-31092). Those comments are all valuable and very helpful for revising and improving our paper, as well as the important guiding significance to our researches.

First, here is our response to the Journal Requirements.

**1. Please ensure that your manuscript meets PLOS ONE's style requirements, including those for file naming.**

**Response:**

First of all, we would like to thank the journal again for their guidance, and we have reworked the manuscript style as requested by the journal.

**2. Thank you for stating the following financial disclosure: "The National Key Research and Development Program of China (2018YFC2002204), Hubei Key Research and Development Program (2022BCA045) and The National Natural Science Foundation of China (81971364 & 82001527)."**

**Please state what role the funders took in the study. If the funders had no role, please state: "The funders had no role in study design, data collection and analysis, decision to publish, or preparation of the manuscript."**

**If this statement is not correct you must amend it as needed.**

**Please include this amended Role of Funder statement in your cover letter; we will change the online submission form on your behalf.**

**Response:**

The above funds are our funders, but **"The funders had no role in study design, data collection and analysis, decision to publish, or preparation of the manuscript. "** And we will explain this again in the cover letter.

**3. PLOS requires an ORCID iD for the corresponding author in Editorial Manager on papers submitted after December 6th, 2016. Please ensure that you have an ORCID iD and that it is validated in Editorial Manager. To do this, go to ‘Update my Information’ (in the upper left-hand corner of the main menu), and click on the Fetch/Validate link next to the ORCID field. This will take you to the ORCID site and allow you to create a new iD or authenticate a pre-existing iD in Editorial Manager. Please see the following video for instructions on linking an ORCID iD to your Editorial Manager account:** [**https://www.youtube.com/watch?v=_xcclfuvtxQ**](https://www.youtube.com/watch?v=_xcclfuvtxQ)

**Response:**

We double-checked our account and found that the corresponding author's ORCID iD exists in the system, as shown below.

**4. Please include captions for your Supporting Information files at the end of your manuscript, and update any in-text citations to match accordingly. Please see our Supporting Information guidelines for more information: http://journals.plos.org/plosone/s/supporting-information.**

**Response:**

We have added the title of the supporting information at the end of the manuscript and updated the references in the manuscript accordingly.

5. We note that Figure 1 in your submission contain [map/satellite] images which may be copyrighted. All PLOS content is published under the Creative Commons Attribution License (CC BY 4.0), which means that the manuscript, images, and Supporting Information files will be freely available online, and any third party is permitted to access, download, copy, distribute, and use these materials in any way, even commercially, with proper attribution. For these reasons, we cannot publish previously copyrighted maps or satellite images created using proprietary data, such as Google software (Google Maps, Street View, and Earth). For more information, see our copyright guidelines: <http://journals.plos.org/plosone/s/licenses-and-copyright>.

**Response:**

Thank you for your reminder that we take copyright issues very seriously, so we immediately went to investigate the source of the map. Since our map was generated by R, we investigated the source of the map in the R package (as shown below) and found that it came from Natural Earth (http://www.naturalearthdata.com/), which is in the public domain and therefore does not require special attribution, but we will make the source of the Figure clear in the manuscript.

6. Please review your reference list to ensure that it is complete and correct. If you have cited papers that have been retracted, please include the rationale for doing so in the manuscript text, or remove these references and replace them with relevant current references. Any changes to the reference list should be mentioned in the rebuttal letter that accompanies your revised manuscript. If you need to cite a retracted article, indicate the article’s retracted status in the References list and also include a citation and full reference for the retraction notice.

**Response:**

We have checked the reference list, and we can now be sure that it is correct.

**Here is a point-by-point response to the comments and concerns of the reviewers.**

**Comment 1: The abstract is written with an appropriate focus.**

**Comment 4: Written Properly.**

**Comment 7: The conclusion is written very well.**

**Response:**

Thank you very much for your recognition of our work, and I believe that with your suggestions, our article will be more rigorous and more valuable.

**Comment 2: The introduction section is poorly written.**

**Response:**

We are very sorry for the poorly written introduction, we have revised the manuscript according to your comments and added the key missing parts you suggested, thank you very much for your pointers.

**Comment 3: In addition to the current contents of the introduction section, add at least two paragraphs in the social context like.**

1. What is the situation of EP in developed, developing, and underdeveloped countries? elaborate.

2. How is it changing over time?

3. Where it is still high from the previous literature.

4. Elaborate if it is declining, then why is your study important?

**Response:**

Your suggestions were meticulous and thoughtful, and we have added to the content of the manuscript.

**Comment 5: Figure resolution is very poor. Please provide high-resolution figures.**

**Response:**

We are very sorry that it affects the readability of the article. The images we uploaded in the system are very clear, but the image quality is compressed when the system automatically generates PDF files, so you can download the original images we uploaded by clicking the download button on the top right corner of the image (as shown in the picture), thank you very much for your patience.

**Comment 6: Add some discussion for underdeveloped and developing countries’ situations.**

**Response:**

As you suggested we have added a discussion of underdeveloped and developing countries to the manuscript.

We are very grateful to the editor and reviewers for giving us this opportunity to revise the manuscript. We have tried our best to improve the manuscript, made some changes to the manuscript, and responded to the reviewers' questions one by one. We have uploaded the ***Manuscript*** and ***Revised Manuscript with Track Changes***. We appreciate for editor and reviewers' warm work earnestly, and hope that the correction will meet with approval. Once again, thank you very much for your comments and suggestions.

Thank you and best regards.

Yours sincerely,

Shufei Zhang

Corresponding author：

Li Hong, Ph.D.

E-mail: dr_hongli@whu.edu.cn
