# Supplementary material for: Global burden and trends of ectopic pregnancy: An observational trend study from 1990 to 2019
Source: PLoS One. 2023 Oct 26;18(10):e0291316. doi: 10.1371/journal.pone.0291316 (PMC10602312; doi:10.1371/journal.pone.0291316)
Supplement: Attachment — Submitted filename: Response to Reviewers.docx [file pone.0291316.s010.docx]

Dear Editors and Reviewers,

Thank you for your letter and for the reviewers' comments concerning our manuscript entitled " Global burden and trends of ectopic pregnancy: An observational trend study from 1990 to 2019" (PONE-D-22-31092R1). Those comments are all valuable and very helpful for revising and improving our paper, as well as the important guiding significance to our research.

**First, here is our response to the Journal Requirements.**

**1. Please review your reference list to ensure that it is complete and correct. If you have cited papers that have been retracted, please include the rationale for doing so in the manuscript text, or remove these references and replace them with relevant current references. Any changes to the reference list should be mentioned in the rebuttal letter that accompanies your revised manuscript. If you need to cite a retracted article, indicate the article’s retracted status in the References list and also include a citation and full reference for the retraction notice.**

**Response:**

Thank you for your patience in reviewing the manuscript, we have checked all the references to ensure that none of them have been withdrawn, and although we have changed some references, we can be sure that the reference list is correct.

**Here are our responses to the reviewers' general comments**

We truly appreciate your suggestions on our manuscript and are very sorry that the article's readability has been reduced due to our writing, so we have tried our best to polish the language in the revised manuscript. As for the statistical issues, we have also made changes and deletions based on your suggestions. We hope the revised manuscript could be acceptable to you.

**Here is a point-by-point response to the comments and concerns of the reviewers.**

**Comments on the introduction:**

**1. The introduction section is poorly written**

**Response:**

We sincerely apologize for our poor writing, and we have carefully and completely rewritten the Introduction section of the manuscript.

**2. There is no need to discuss diagnosis and treatment in the introduction**

**Response:**

Thank you very much for your suggestions, we couldn't agree with you more, so we have removed the relevant content and have carefully rewritten this section, and we hope that these changes will fulfill your requirements for the manuscript.

**Comments on the Methods:**

**1. Study data was obtained from The Global Burden of Disease Study 2019 was modeled by the Institute for Health Metrics and Evaluation (IHME). SDI data obtained from Global Health Data Exchange (GHDx)**

**Response:**

First of all, thank you very much for your suggestions, we have added this important information to the revised manuscript.

**Line 62-64**, “Study data was obtained from GBD 2019 was modeled by the Institute for Health Metrics and Evaluation (IHME)” was added.

**Line 70-71**, “SDI data was obtained from Global Health Data Exchange (GHDx)” was added.

**2. Global Burden of Disease Study 2019 should be introduced in details**

**Response:**

We are very sorry for our omission of this important section, so we have added an introduction to the Global Burden of Disease Study 2019 in the revised manuscript.

**Line 53-55**, “Explore results from the 2019 Global Burden of Disease study (GBD 2019), which published epidemiological data related to 369 diseases/injuries and 286 causes of death, covered EP in 204 countries and territories from 1990 to 2019” was added.

References:

<http://doi.org/10.1016/j.ajp.2023.103677>

**3. SDI classes should be explained**

**Response:**

We are very sorry that our description of SDI was not clear, so we have made it explicit in the revised manuscript.

**Line 65-69**, “The Socio-demographic Index (SDI) is a composite indicator of development status strongly correlated with health outcomes. It is the geometric mean of 0 to 1 indices of total fertility rate under the age of 25, mean education for those ages 15 and older and lag distributed income per capita. As a composite, a location with an SDI of 0 would have a theoretical minimum level of development relevant to health, while a location with an SDI of 1 would have a theoretical maximum level” was added.

References:

<https://ghdx.healthdata.org/sites/default/files/record-attached-files/IHME_GBD_2019_SDI_1950_2019_INFO_SHEET_Y2021M08D16.PDF>

**4. How is EP defined in GBD protocols?**

**Response:**

Thank you very much for your suggestion regarding the definition of ectopic pregnancy, which is a very important issue. We have managed to find the GBD definition of ectopic pregnancy after a careful search of the sources and have added it to the revised manuscript.

**Line 61**, “In GBD, EP is defined as pregnancy occurring outside of the uterus.” was added.

References:

<https://www.healthdata.org/results/gbd_summaries/2019/ectopic-pregnancy-level-4-cause>

**5. Age-standardized incidence rate, mortality rate and DALY rate were defined in GBD, why haven't researchers used them and recalculated?**

**Response:**

Thank you very much for your suggestion, we have calculated the Age-standardized incidence rate, mortality rate, and DALY rate in the table and abbreviated them as ASIR, ASDR, and AS-DALYs in the Method section and have used them for calculation and visualization in the results.

**6. “EAPCs were calculated using a linear regression model as follows: ln (ASR) = α + β x + ε,” is incorrect**

**Response:**

Thank you for your correction, we have corrected it in the revised manuscript.

**Line 77-78**, “EAPCs were calculated using a linear regression model as follows: ln (ASR) = α + β x + ε” was corrected as “The EAPC is calculated by fitting the linear regression line: Y = α + βx + ε, where y represents ln(ASR) and x refers to the calendar year.”.

**7. R language version 4.2.1 is incorrect, R software is correct**

**Response:**

Thanks again for the correction; we have also corrected it in the revised manuscript.

**Line 87**, “R language” was corrected as “R software”.

**Comments on the Results:**

**1. 3.1 Distribution and trends in the incidence rate of EP by age or year???**

**Response:**

Thank you very much for your patience, we have corrected several titles that did not make sense in the revised manuscript.

**Line 91**, “3.1 Distribution and trends in the incidence rate of EP by age or year” was corrected as “3.1 Distribution and trends in the incidence rate of EP”.

**Line 115**, “3.2 Distribution and trends in the DALYs rate of EP by age or year” was corrected as “3.2 Distribution and trends in the DALYs rate of EP”.

**Line 133**, “3.3 Distribution and trends in the Death rate of EP by age or year” was corrected as “3.3 Distribution and trends in the Death rate of EP”.

**2. Figures resolution is very poor. Please provide high-resolution figures**

**Response:**

We are very sorry that the unclear figures have caused problems for your review, we have remade and uploaded all the figures and ensured their clarity. However, the journal requires that figures should not exceed 10M in size, so there may be some figures with crowded and unclear details.

**3. Figure 2 is unclear**

**Response:**

Thanks for your suggestion, we have recreated and uploaded a clear figure 2.

**4. the 0-14 and e 55+ years age groups should be excluded**

**Response:**

We acknowledge that it may be unreasonable to discuss ectopic pregnancies in women under 14 years old or over 55 years old, therefore, we excluded cases in these two age groups and re-visualized them, as shown in Figure 2, S4 Figure, and S5 Figure.

**5. the results of EP-related risk factors not well presented**

**Response:**

Thank you for your suggestion and we recognize the limitations of this study in terms of the risk factors associated with ectopic pregnancy. This is because GBD 2019 contains only one risk factor for ectopic pregnancy, iron deficiency, and therefore we have only explored it in the manuscript. We have also rewritten this section in the revised manuscript, and we hope that these changes will help the reader better understand the contents of the manuscript.

**Comments on the discussion:**

**1. discussion section is weak**

**Response:**

Thank you very much for your patient suggestions, we have carefully rewritten the discussion section of the manuscript. The revised manuscript provides a comprehensive analysis of the condition of ectopic pregnancy on several levels.

**2. advantage and limitation were missed**

**Response:**

We recognize that an exploration of the advantages and limitations of this study was missing from our manuscript, so in the revised manuscript, we have added this section. Thanks to your suggestions, this makes our manuscript better.

**Line 271-286**, “The present study has several notable strengths. Firstly, it provides a comprehensive analysis of EP morbidity, mortality, and DALYs over a period of 30 years (1990-2019), offering a valuable overview of the global burden of EP. Additionally, the study examines the influencing factors associated with EP by comparing its conditions in different SDI and age groups, thereby providing new insights and directions for further research on EP. This study is the first-ever global epidemiological study on EP, making it an important reference for EP management. However, despite these strengths, there are some limitations that should be acknowledged. Firstly, the study relies on data from the GBD estimation, which may introduce uncertainty due to variations in data availability across countries and regions. Moreover, the lack of a comprehensive EP surveillance system in certain low and Middle SDI countries/regions further contributes to potential data limitations. Secondly, the study does not differentiate between subtypes or pathological types of EP in the GBD analysis, hindering a more detailed disease analysis. Furthermore, it is worth noting that only one EP-related risk factor, iron deficiency, was included in the study, while common factors such as Chlamydia trachomatis infections and pelvic inflammatory disease were not identified as significant risk factors. This could be considered an incomplete and potentially biased representation of EP risk factors. However, modifying the availability of such data can be challenging.” was added.

We are very grateful to the editor and reviewers for giving us this opportunity to revise the manuscript. We have tried our best to improve the manuscript, made some changes to the manuscript, and responded to the reviewers' questions one by one. We have uploaded the ***Manuscript*** and ***Revised Manuscript with Track Changes***. We appreciate for editor and reviewers' warm work earnestly and hope that the correction will meet with approval. Once again, thank you very much for your comments and suggestions.

Thank you and best regards.

Yours sincerely,

Shufei Zhang

Corresponding author：

Li Hong, Ph.D.

E-mail: dr_hongli@whu.edu.cn
